# From the Phenomenology to the Mechanisms of Consciousness: Integrated Information Theory 3.0

**DOI:** 10.1371/journal.pcbi.1003588

**Published:** 2014-05-08

**Authors:** Masafumi Oizumi, Larissa Albantakis, Giulio Tononi

**Affiliations:** 1 Department of Psychiatry, University of Wisconsin, Madison, Wisconsin, United States of America; 2 RIKEN Brain Science Institute, Wako-shi, Saitama, Japan; Indiana University, United States of America

## Abstract

This paper presents Integrated Information Theory (IIT) of consciousness 3.0, which incorporates several advances over previous formulations. IIT starts from phenomenological axioms: information says that each experience is specific – it is what it is by how it differs from alternative experiences; integration says that it is unified – irreducible to non-interdependent components; exclusion says that it has unique borders and a particular spatio-temporal grain. These axioms are formalized into postulates that prescribe how physical mechanisms, such as neurons or logic gates, must be configured to generate experience (phenomenology). The postulates are used to define intrinsic information as “differences that make a difference” within a system, and integrated information as information specified by a whole that cannot be reduced to that specified by its parts. By applying the postulates both at the level of individual mechanisms and at the level of systems of mechanisms, IIT arrives at an identity: an experience is a maximally irreducible conceptual structure (*MICS*, a constellation of concepts in qualia space), and the set of elements that generates it constitutes a *complex*. According to IIT, a MICS specifies the quality of an experience and integrated information Φ^Max^ its quantity. From the theory follow several results, including: a system of mechanisms may condense into a major complex and non-overlapping minor complexes; the concepts that specify the quality of an experience are always about the complex itself and relate only indirectly to the external environment; anatomical connectivity influences complexes and associated MICS; a complex can generate a MICS even if its elements are inactive; simple systems can be minimally conscious; complicated systems can be unconscious; there can be true “zombies” – unconscious feed-forward systems that are functionally equivalent to conscious complexes.

## Author Summary

Integrated information theory (IIT) approaches the relationship between consciousness and its physical substrate by first identifying the fundamental properties of experience itself: existence, composition, information, integration, and exclusion. IIT then postulates that the physical substrate of consciousness must satisfy these very properties. We develop a detailed mathematical framework in which composition, information, integration, and exclusion are defined precisely and made operational. This allows us to establish to what extent simple systems of mechanisms, such as logic gates or neuron-like elements, can form complexes that can account for the fundamental properties of consciousness. Based on this principled approach, we show that IIT can explain many known facts about consciousness and the brain, leads to specific predictions, and allows us to infer, at least in principle, both the quantity and quality of consciousness for systems whose causal structure is known. For example, we show that some simple systems can be minimally conscious, some complicated systems can be unconscious, and two different systems can be functionally equivalent, yet one is conscious and the other one is not.

## Introduction

Understanding consciousness requires not only empirical studies of its neural correlates, but also a principled theoretical approach that can provide explanatory, inferential, and predictive power. For example, why is consciousness generated by the corticothalamic system – or at least some parts of it, but not by the cerebellum, despite the latter having even more neurons? Why does consciousness fade early in sleep, although the brain remains active? Why is it lost during generalized seizures, when neural activity is intense and synchronous? And why is there no direct contribution to consciousness from neural activity within sensory and motor pathways, or within neural circuits looping out of the cortex into subcortical structures and back, despite their manifest ability to influence the content of experience? Explaining these facts in a parsimonious manner calls for a theory of consciousness. (Below, consciousness, experience, and phenomenology are taken as being synonymous).

A theory is also needed for making inferences in difficult or ambiguous cases. For example, is a newborn baby conscious, how much, and of what? Or an animal like a bat, a lizard, a fruit fly? In such cases, one cannot resort to verbal reports to establish the presence and nature of consciousness, or to the neural correlates of consciousness as established in healthy adults. The inadequacy of behavioral assessments of consciousness is also evident in many brain-damaged patients, who cannot communicate, and whose brain may be working in ways that are hard to interpret. Is a clinically vegetative patient showing an island of residual, near-normal brain activity in just one region of the cortex conscious, how much, and of what? Or is nobody home? Or again, consider machines, which are becoming more and more sophisticated at reproducing human cognitive abilities and at interacting profitably with us. Some machines can learn to categorize objects such as faces, places, animals, and so on, as well if not better than humans [1], or can answer difficult questions better than humans [2], [3]. Are such machines approaching our level of consciousness? If not, what are they missing, and what does it take to build a machine that is actually conscious? Clearly, only a theory - one that says what consciousness is and how it can be generated - can hope to offer a combination of explanatory, inferential, and predictive power starting from a few basic principles, and provide a way to quantify both the level of consciousness and its content.

Integrated information theory (IIT) is an attempt to characterize consciousness mathematically both in quantity and in quality [4]–[6]. IIT starts from the fundamental properties of the phenomenology of consciousness, which are identified as *axioms* of consciousness. Then, IIT translates these axioms into *postulates*, which specify which conditions must be satisfied by physical mechanisms, such as neurons and their connections, to account for the phenomenology of consciousness. It must be emphasized that taking the phenomenology of consciousness as primary, and asking how it can be implemented by physical mechanisms, is the opposite of the approach usually taken in neuroscience: start from neural mechanisms in the brain, and ask under what conditions they give rise to consciousness, as assessed by behavioral reports [7]–[10]. While identifying the “neural correlates of consciousness” is undoubtedly important [8], it is hard to see how it could ever lead to a satisfactory explanation of what consciousness is and how it comes about [11].

As will be illustrated below, IIT offers a way to analyze systems of mechanisms to determine if they are properly structured to give rise to consciousness, how much of it, and of which kind. As reviewed previously [4], [5], [12], [13], the fundamental principles of IIT, such as integration and differentiation, can provide a parsimonious explanation for many neuroanatomical, neurophysiological, and neuropsychological findings concerning the neural substrate of consciousness. Moreover, IIT leads to experimental predictions, for instance that the loss and recovery of consciousness should be associated with the breakdown and recovery of information integration. This prediction has been confirmed using transcranial magnetic stimulation in combination with high-density electroencephalography in several different conditions characterized by loss of consciousness, such as deep sleep, general anesthesia obtained with several different agents, and in brain damaged patients (vegetative, minimally conscious, emerging from minimal consciousness, locked-in [14]). Furthermore, IIT has inspired theoretically motivated measures of the level of consciousness that have been applied to human and animal data (e.g. [14], see also [15] for a related attempt to measure the level of consciousness based on symbolic mutual information).

While the central assumptions of IIT have remained the same, its theoretical apparatus has undergone various developments over the years. The original formulation, which may be called IIT 1.0, introduced the essential notions including causal measures of the quantity and quality of consciousness. However, to simplify the analysis, IIT 1.0 dealt exclusively with stationary systems [4] (see also [16]). The next formulation, which will be called IIT 2.0 [5], [17], [18] applied the same notions on a state-dependent basis: it showed how integrated information could be calculated in a top-down manner for a system of mechanisms in a state [17] and suggested a way to characterize the quality of an experience by considering its sub-mechanisms [18]. The formulation presented below, and the new results that follow from it, represent a substantial advance at several different levels, hence IIT 3.0 (see also [6]). Nevertheless, this article is presented independently of previous “releases” for readers new to IIT. For those readers who may have followed the evolution of IIT, the main advances are summarized in the Supplementary Material (Text S1).

In what follows, we first present the axioms and the postulates of IIT. We then provide the mathematical formalism and motivating examples for each of the postulates. The key constructs of IIT are introduced first at the level of individual mechanisms, which can be taken to represent physical objects such as logic gates or neurons, then at the level of systems of mechanisms, such as computers or neural architectures. The Models section ends by presenting the central identity proposed by IIT, according to which the quality and quantity of an experience is completely specified by a maximally irreducible conceptual structure (MICS) and the associated value of integrated information Φ^Max^. The Results/Discussion section presents several new results that follow directly from IIT, including the condensation of systems of mechanisms into main complexes and minor complexes; examples of simple systems that are minimally conscious and of complicated systems that are not; an example of an unconscious feed-forward system that is functionally equivalent to a conscious complex; and finally, an example showing that concepts within a complex are self-referential and relate only indirectly to the external environment.

## Models

### Axioms, postulates, and identities

The main tenets of IIT can be presented as a set of phenomenological axioms, ontological postulates, and identities. While the terms “axioms” and “postulates” are often used interchangeably, we follow the classical tradition according to which an “axiom” is a self-evident truth, whereas a “postulate” is an unproven assumption that can serve as the basis for logic or heuristics. Here the distinction takes on an even stronger meaning: axioms are self-evident truths about consciousness – the only truths that, with Descartes, cannot be doubted and do not need proof (experience exists, it is irreducible etc.). Postulates instead are assumptions about the physical world and specifically about the physical substrates of consciousness (mechanisms must exist, be irreducible, etc.), which can be formalized and form the basis of the mathematical framework of IIT.

#### Axioms

The central axioms, which are taken to be immediately evident, are as follows:

- Existence: Consciousness exists – it is an undeniable aspect of reality. Paraphrasing Descartes, “I experience therefore I am”.
- Composition: Consciousness is compositional (structured): each experience consists of multiple aspects in various combinations. Within the same experience, one can see, for example, left and right, red and blue, a triangle and a square, a red triangle on the left, a blue square on the right, and so on.
- Information: Consciousness is informative: each experience differs in its particular way from other possible experiences. Thus, an experience of pure darkness is what it is by differing, in its particular way, from an immense number of other possible experiences. A small subset of these possible experiences includes, for example, all the frames of all possible movies.
- Integration: Consciousness is integrated: each experience is (strongly) irreducible to non-interdependent components. Thus, experiencing the word “SONO” written in the middle of a blank page is irreducible to an experience of the word “SO” at the right border of a half-page, plus an experience of the word “NO” on the left border of another half page – the experience is whole. Similarly, seeing a red triangle is irreducible to seeing a triangle but no red color, plus a red patch but no triangle.
- Exclusion: Consciousness is exclusive: each experience excludes all others – at any given time there is only one experience having its full content, rather than a superposition of multiple partial experiences; each experience has definite borders – certain things can be experienced and others cannot; each experience has a particular spatial and temporal grain – it flows at a particular speed, and it has a certain resolution such that some distinctions are possible and finer or coarser distinctions are not.

#### Postulates

To parallel the phenomenological axioms, IIT posits a set of postulates. These list the properties physical systems must satisfy in order to generate experience.

- Existence: Mechanisms in a state exist. A system is a set of mechanisms.
- Composition: Elementary mechanisms can be combined into higher order ones.

The next three postulates, information, integration, and exclusion, apply both to individual mechanisms and to systems of mechanisms.

#### Mechanisms

- Information: A mechanism can contribute to consciousness only if it specifies “differences that make a difference” within a system. That is, a mechanism in a state generates information only if it constrains the states of a system that can be its possible causes and effects – its *cause-effect repertoire*. The more selective the possible causes and effects, the higher the *cause-effect information cei* specified by the mechanism.
- Integration: A mechanism can contribute to consciousness only if it specifies a cause-effect repertoire (information) that is *irreducible* to independent components. *Integration/irreducibility φ* is assessed by partitioning the mechanism and measuring what difference this makes to its cause-effect repertoire.
- Exclusion: A mechanism can contribute to consciousness at most one cause-effect repertoire, the one having the maximum value of *integration/irreducibility φ* ^Max^. This is its *maximally irreducible* cause-effect repertoire (MICE, or *quale sensu stricto* (in the narrow sense of the word, [5])). If the MICE exists, the mechanism constitutes a *concept*.

#### Systems of mechanisms

- Information: A set of elements can be conscious only if its mechanisms specify a set of “differences that make a difference” to the set – i.e. a *conceptual structure*. A conceptual structure is a *constellation* of points in concept space, where each axis is a possible past/future state of the set of elements, and each point is a concept specifying differences that make a difference within the set. The higher the number of different concepts and their *φ* ^Max^ value, the higher the *conceptual information CI* that specifies a particular constellation and distinguishes it from other possible constellations.
- Integration: A set of elements can be conscious only if its mechanisms specify a conceptual structure that is *irreducible* to non-interdependent components (strong integration). *Strong integration/irreducibility* Φ is assessed by partitioning the set of elements into subsets with unidirectional cuts.
- Exclusion: Of all overlapping sets of elements, only one set can be conscious – the one whose mechanisms specify a conceptual structure that is *maximally irreducible (MICS)* to independent components. A local maximum of integrated information Φ^Max^ (over elements, space, and time) is called a *complex*.

#### Identities

Finally, according to IIT, there is an identity between phenomenological properties of experience and informational/causal properties of physical systems (see [11] and [19] for the importance of identities for the mind-body problem). The central identity is the following:

The maximally irreducible conceptual structure (*MICS*) generated by a complex of elements is identical to its experience. The constellation of concepts of the MICS completely specifies the quality of the experience (its *quale “sensu lato”* (in the broad sense of the term [5])). Its irreducibility Φ^Max^ specifies its quantity. The maximally irreducible cause-effect repertoire (MICE) of each concept within a MICS specifies what the concept is about (what it contributes to the quality of the experience, i.e. its *quale sensu stricto* (in the narrow sense of the term)), while its value of irreducibility *φ* ^Max^ specifies how much the concept is present in the experience. An experience is thus an *intrinsic property* of a complex of mechanisms in a state. In other words, the maximally irreducible conceptual structure specified by a complex exists intrinsically (from its own intrinsic perspective), without the need for an external observer.

### Mechanisms

In what follows, we consider simple systems that can be used to illustrate the postulates of IIT. In the first part, we apply the postulates of IIT at the level of *individual mechanisms*. We show that an individual mechanism generates information by specifying both selective causes and effects (information), that it needs to be irreducible to independent components (integration), and that only the most irreducible cause-effect repertoire of each mechanism should be considered (exclusion). This allows us to introduce the notion of a *concept*: the maximally irreducible cause-effect repertoire of a mechanism.

In the next part, we consider the postulates of IIT at the level of *systems of mechanisms*, and show how the requirements for information, integration, and exclusion can be satisfied at the system level. This allows us to introduce the notion of a *complex* – a maximally integrated set of elements – and of a *quale* – the maximally irreducible conceptual structure (MICS) it generates. Altogether, these two sections show how to assess in a step-by-step, bottom up manner, whether a system generates a maximally integrated conceptual structure and how the latter can be characterized in full. A summary of the key concepts and associated measures is provided as a reference in Table 1 and Box 1.

**Table 1:** Key concepts and measures of IIT.

MECHANISM	SYSTEM OF MECHANISMS
**Information**	
Only mechanisms that specify differences that make a difference within a system count	
**Cause-effect information** (*cei*): How a mechanism in a state specifies the probability of past and future states of a set of elements (cause-effect repertoires)	**Conceptual information** (*CI*): How a set of mechanisms specifies the probability of past and future states of the set (conceptual structure)
**Integration**	
Only information that is irreducible to independent components counts	
**Integrated information (** ***φ*** **, “small phi”):** How irreducible the cause-effect repertoire specified by a mechanism is compared to its minimum information partition (MIP)	**Integrated conceptual information (Φ, “big phi”):** How irreducible the conceptual structure specified by a set of mechanism is compared to its minimum information partition (MIP)
**Exclusion**	
Only maxima of integrated information count (over elements, space, time)	
**Concept (** ***φ*** *^Max^* **):** A mechanism that specifies a maximally irreducible cause-effect repertoire (MICE or quale “sensu stricto”)	**Complex (Φ** ***^Max^*** **):** A set of elements whose mechanisms specify a maximally irreducible conceptual structure (MICS or quale “sensu lato”)

**Box 1. Glossary**

**Axiom:** Self-evident truth about consciousness (experience exists, it is irreducible etc.). The only truths that, with Descartes, cannot be doubted and do not need proof. They are existence, composition, information, integration, and exclusion (see text).

**Background conditions:** Fixed external constrains on a candidate set of elements. Past and current state of the elements outside the candidate set are fixed to their actual values.

**Candidate set:** The set of elements under consideration. Elements inside the candidate set are perturbed into all their possible states to obtain the TPM of the candidate set.

**Cause-effect repertoire:** The probability distribution of potential past and future states of a system as constrained by a mechanism in its current state.

**Cause-effect information (** ***cei*** **):** The amount of information specified by a mechanism in a state, measured as the minimum of cause information (*ci*) and effect information (*ei*).

**Cause information (** ***ci*** **) and effect information (** ***ei*** **):** Information about the past and the future, which is measured as the distance between the cause repertoire and the unconstrained cause repertoire (same on the effect side).

**Complex:** A set of elements within a system that generates a local maximum of integrated conceptual information Φ^Max^. Only a complex exists as an entity from its own intrinsic perspective.

**Concept:** A set of elements within a system and the maximally irreducible cause-effect repertoire it specifies, with its associated value of integrated information *φ* ^Max^. The concept expresses the causal role of a mechanism within a complex.

**Conceptual structure, constellation of concepts (** ***C*** **):** A conceptual structure is the set of all concepts specified by a candidate set with their respective *φ* ^Max^ values, which can be plotted as a constellation in concept space.

**Conceptual information (** ***CI*** **):** A measure of how many different concepts are generated by a system of elements. *CI* is quantified by the distance D between the constellation of concepts and the “null” concept, the unconstrained cause-effect repertoire *p^uc^*.

**Concept space:** Concept space is a high dimensional space with one axis for each possible past and future state of the system in which a conceptual structure can be represented.

**Distance (D):** In IIT 3.0, the Wasserstein distance, also known as earth mover's distance (EMD). It specifies the metric of concept space and thus the distance between probability distributions (*φ*) and between constellations of concepts (Φ).

**Integrated conceptual information (Φ):** Conceptual information that is generated by a system above and beyond the conceptual information generated by its (minimal) parts. Φ measures the integration or irreducibility of a constellation of concepts (integration at the system level).

**Integrated information (** ***φ*** **):** Information that is generated by a mechanism above and beyond the information generated by its (minimal) parts. *φ* measures the integration or irreducibility of mechanisms (integration at the mechanism level).

**Intrinsic information:** Differences that make a difference within a system.

**Mechanism:** Any subsystem of a system, including the system itself, that has a causal role within the system, for example, a neuron in the brain, or a logic gate in a computer.

**MICE (maximally irreducible cause-effect repertoire):** The cause-effect repertoire of a concept, i.e., the cause-effect repertoire that generates a maximum of integrated information *φ* among all possible purviews.

**MICS (maximally irreducible conceptual structure):** The conceptual structure generated by a complex in a state that corresponds to a local maximum of integrated conceptual information Φ^Max^ (synonymous with “quale” or “constellation” in “qualia space”).

**MIP (minimum information partition):** The partition that makes the least difference (in other words, the minimum “difference” partition).

**Null concept:** The unconstrained cause-effect repertoire *p^uc^* of the candidate set, with *φ* = 0.

**Partition:** Division of a set of elements into causally/informationally independent parts, performed by noising the connections between the parts.

**Power set:** The set of all subsets of a candidate set of elements.

**Postulates:** Assumptions, derived from axioms, about the physical substrates of consciousness (mechanisms must have causal power, be irreducible, etc.), which can be formalized and form the basis of the mathematical framework of IIT. They are existence, composition, information, integration, and exclusion (see text).

**Purview:** Any set of elements of a candidate set over which the cause and effect repertoires of a mechanism in a state are calculated.

**Quale:** The conceptual structure generated by a complex in a state that corresponds to a local maximum of integrated conceptual information Φ^Max^ (synonymous with “MICS” or “constellation” in “qualia space”).

**Qualia space:** If a set of elements forms a complex, its concept space is called qualia space.

**System:** A set of elements/mechanisms.

**TPM (transition probability matrix):** A matrix that specifies the probability with which any state of a system transitions to any other system state. The TPM is determined by the mechanisms of a system and obtained by perturbing the system into all its possible states.

**Unconstrained repertoire (** ***p^uc^*** **)**: The probability distribution of potential past and future system states without constraints due to any mechanism in a state. The unconstrained cause repertoire is the uniform distribution of system states. The unconstrained effect repertoire is obtained by assuming unconstrained inputs to all system elements.

#### Existence

The existence postulate, the “zeroth” postulate of IIT, claims that mechanisms in a state exist. Within the present framework, “mechanism” simply denotes anything having a causal role within a system, for example, a neuron in the brain, or a logic gate in a computer. In principle, mechanisms might be characterized at various spatio-temporal scales, down to the micro-physical level, although for any given system there will be a scale at which causal interactions are strongest [20]. In what follows, we consider systems in which the elementary mechanisms are discrete logic gates or linear threshold units (Text S2) and assume that these mechanisms are the ones mediating the strongest causal interactions.

**Figure 1:**
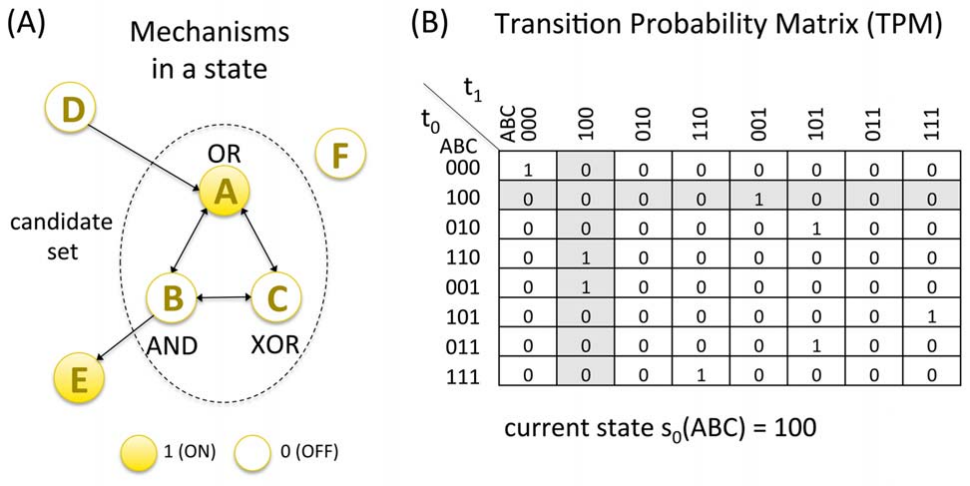
Existence: Mechanisms in a state having causal power. (A) The dotted circle indicates elements *ABC* as the candidate set of mechanisms. Elements outside the candidate set (*D*, *E*, *F*) are taken as background conditions (external constraints). The logic gates *A*, *B*, and *C* are represented as is customary in neural circuits rather than electronic circuits. The arrows indicate directed connections between the elements. (B) The set's mechanisms *ABC* determine the transition probability matrix (TPM) of the set under the background conditions of *DEF* (here *DEF*(*t*_−1_) = *DEF*(*t*_0_) = 010). With element *D* fixed to *D* = 0, element *A*, for instance, receives inputs from *B* and *C* and outputs to *B* and *C*. The OR gate *A* is on (1) if either *B*, or *C*, or both were on at the last time step, and off (0) if *BC* was 00. Filled circles denote that the state of an element is ‘1’, open circles indicate that the state of an element is ‘0’. The current state of *ABC* is 100.

Figure 1A shows the example system *ABCDEF*, which includes three logic gate mechanisms, OR, AND, XOR, which will be used to illustrate the postulates of IIT throughout the Model section. The dotted circle indicates that the particular set of elements *ABC* is going to be considered as a “candidate set” for IIT analysis, whereas the remaining elements *D*,*E*,*F* are considered external and treated as background conditions (Text S2).

The mechanisms of *ABC* determine the transition probability matrix (TPM) of the candidate set, which specifies the probability with which any state of the set *ABC* transitions into any other state under the background conditions of elements DEF, here 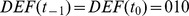 (Figure 1B). In this case, since the system is deterministic, the values in the TPM are 0 or 1, but non-deterministic systems can also be considered. In this example, at the current time step 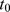, the mechanisms are in state 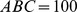. The TPM specifies which past states could have led to the current state 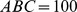 (the shaded column in Figure 1B) and which future states it could go to (shaded row in Figure 1B), out of all possible states of the set.

#### Composition

**Figure 2:**
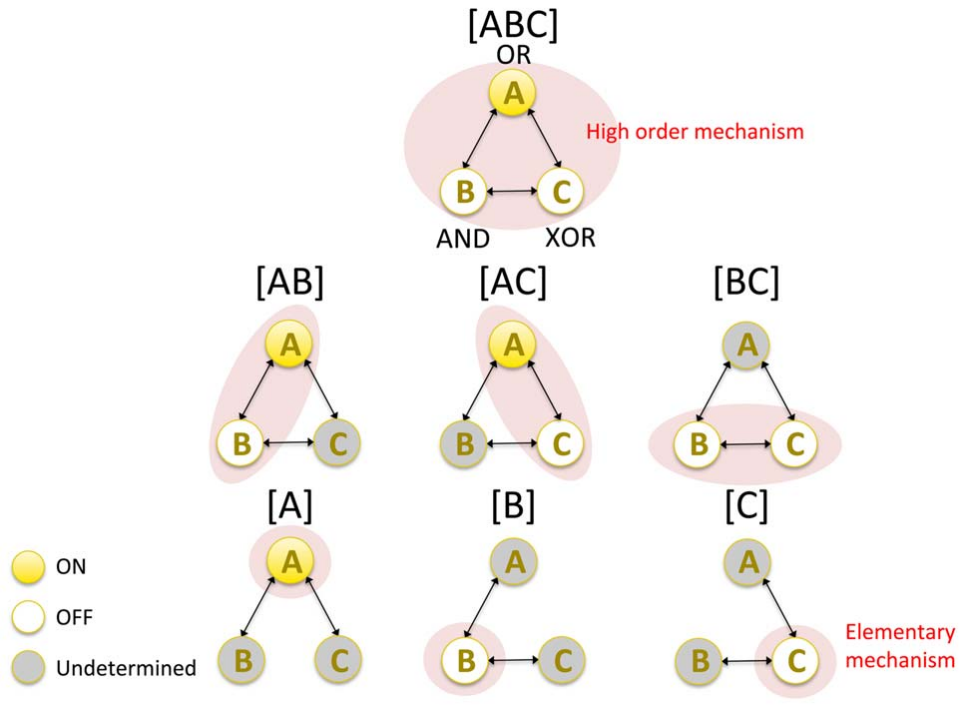
Composition: Higher order mechanisms can be composed by combining elementary mechanisms. The set *ABC* has 3 elementary mechanisms *A*, *B*, and *C* (at the bottom). Second-order mechanisms *AB*, *AC*, and *BC* are shown in the middle row and the third-order mechanism *ABC* (corresponding to the full set) is shown at the top. Altogether, the figure indicates the *power set* of possible mechanisms in set *ABC*. In the figure, each mechanism is highlighted by a red shaded area. The current state of the elements inside the candidate set but outside of a mechanism is undetermined for the mechanism under consideration.

The composition postulate states that elementary mechanisms can be structured, forming higher order mechanisms in various combinations. In Figure 2, *A*, *B*, and *C* are the elementary (first-order) mechanisms. By combining them, higher order mechanisms can be constructed. Pairs of elements form second-order mechanisms (*AB*, *AC*, *BC*), and all elements together form the third-order mechanism *ABC*. A red area highlights the respective mechanisms in Figure 2. The elements inside the candidate set, but outside the mechanism under consideration, are treated as independent noise sources (Text S2). Altogether, the elementary mechanisms and their combinations form the *power set* of possible mechanisms.

#### Information: Cause-effect repertoires and cause-effect information (*cei*)

In IIT, information is meant to capture the “differences that make a difference” from the perspective of the system itself – and is therefore both causal and intrinsic. These and other features distinguish this “intrinsic” notion of information from the “extrinsic”, Shannon notion (see Text S3; cf. [21]–[23] for related approaches to information and causation in networks).

**Figure 3:**
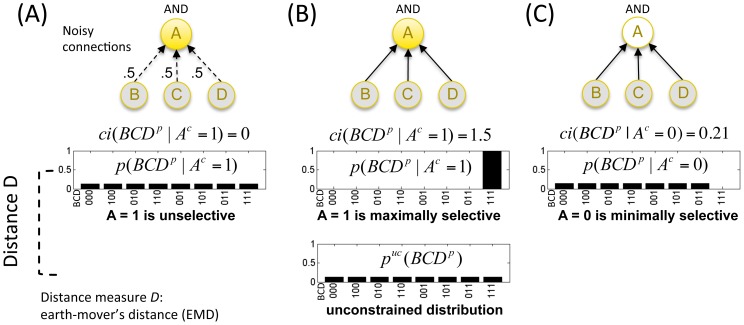
Information requires selectivity. A mechanism generates information to the extent that it selectively constrains a system's past states. Element 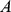 constrains the past states of 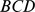 depending on its mechanism (AND gate) and its current state. The constrained distribution of past states is called *A*'s *cause repertoire*. (A) The connections between 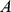 and 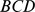 are noisy. *A*'s cause repertoire is thus unselective, since 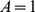 could have followed from any state of 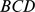 with equal probability. (B) In the case of deterministic connections and current state 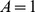, *A*'s cause repertoire is maximally selective, because all states except 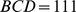 are ruled out as possible causes of 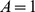. (C) In the case of deterministic connections and current state 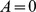, *A*'s cause repertoire is much less selective than for 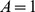, because only state 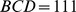 is ruled out as a possible cause of 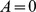.

Information as “differences that make a difference” to a system from its intrinsic perspective can be quantified by considering how a mechanism in its current state 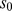 constrains the system's potential past and future states. Figure 3 illustrates how a mechanism *A* constrains the past states of 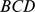 more or less *selectively* depending on its input/output function and state. 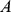 is an AND gate of the inputs from 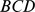. The constrained distribution of past states is called *A*'s *cause repertoire*. In Figure 3A the connections between 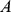 and *BCD* are substituted by noise. Therefore, the current state of *A* cannot specify anything about the past state of *BCD*, the cause repertoire is identical to the unconstrained distribution (unselective), and *A* generates no information. By contrast, when the connections between *A* and *BCD* are deterministic and *A* is on (*A* = 1), the past state of *BCD* is fully constrained, since the only compatible past state is *BCD* = 111 (Figure 3B). In this case, the cause repertoire is maximally selective, corresponding to high information. On the other hand, when 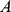 is off (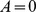, Figure 3C), the cause repertoire is less selective, because only 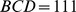 is ruled out, corresponding to less information.

**Figure 4:**
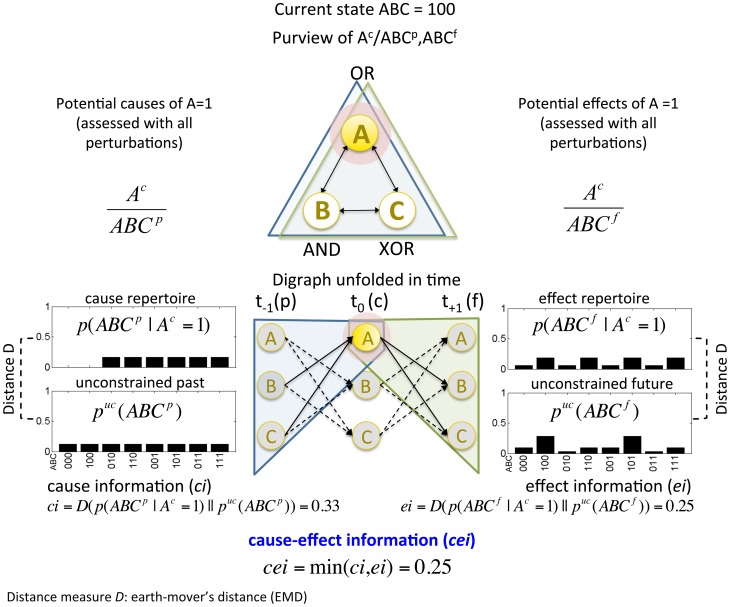
Information: “Differences that make a difference to a system from its own intrinsic perspective.” A mechanism generates information by constraining the system's past and future states. (Top) The candidate set 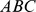 consisting of OR, AND, and XOR gates is shown in its current state 100. We consider the purview of mechanism 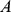, highlighted in red, over the set 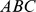 in the past (blue) and in the future (green). (Bottom center) The same network is displayed unfolded over three time steps, from 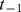 (past), 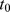 (current) to 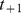 (future). Gray-filled circles are undetermined states. The current state of mechanism *A* constrains the possible past and future system states compared to the unconstrained past and future distributions 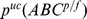. For example, 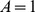 rules out the two states where 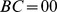 as potential causes. The constrained distribution of past states is *A*'s cause repertoire (left). The constrained distribution of future states is *A*'s effect repertoire (right). Cause information (*ci*) is quantified by measuring the distance *D* between the cause repertoire and the unconstrained past repertoire 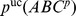; effect information (*ei*) is quantified by measuring the distance *D* between the effect repertoire and the unconstrained future repertoire 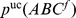. Note that the unconstrained future repertoire 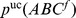 is not simply the uniform distribution, but corresponds to the distribution of future system states with unconstrained inputs to each element. Cause-effect information (*cei*) is then defined as the minimum of *ci* and *ei*.

Figure 4 illustrates how element 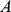 in state 1 constrains the past states (left) and future states (right) of the candidate set 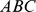. The probability distribution of past states that could have been potential causes of 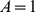 is its cause repertoire 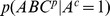. The probability distribution of future states that could be potential effects of 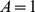 is called *effect repertoire* 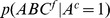. Here, the superscripts ^p^, ^c^, and ^f^ stand for past, current, and future, respectively. The set of elements over which the cause and effect repertoires of a mechanism are calculated is called its *purview*. Figure 4 shows the cause and effect repertoire of mechanism 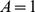 over its purview 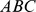 (the full set) in the past and future, labeled 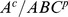 and 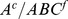. If the purview is not over the full set, the elements outside of the purview are unconstrained (see Text S2 for details on the calculation).

The amount of information that 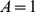 specifies about the past, its cause information (*ci*), is measured as the distance *D* between the cause repertoire 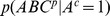 and the unconstrained past repertoire 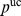. For the purview 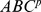:
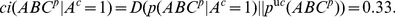
(1)

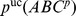
 corresponds to the cause repertoire in the absence of any constraints on the set's output states due to its mechanisms, which is the uniform distribution.

Just like cause information (*ci*), effect information (*ei*) of *A* = 1 is quantified as the distance between the effect repertoire of *A* and the unconstrained future repertoire 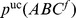:
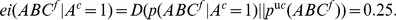
(2)


As can be seen in Figure 4 (right), the unconstrained future repertoire 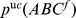 is not simply the uniform distribution of future system states. While 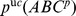 corresponds to the distribution of past system states with unconstrained outputs, 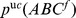 corresponds to the distribution of future system states with unconstrained inputs. Therefore, 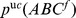 is obtained by perturbing the inputs to each element into all possible states. As an example, the unconstrained future repertoire of element *A*, being an OR gate, is 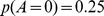 and 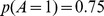, which is obtained by perturbing the inputs of *A* into all possible states 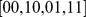.

To quantify differences that make a difference, the distance *D* between two probability distributions is evaluated using the earth mover's distance (EMD) [24], which quantifies how much two distributions differ by taking into account the distance between system states. This is important because, from the intrinsic perspective of the system, it should make a difference if two system elements, rather than just one, differ in their state (see Text S2 for details on the EMD and a discussion of EMD as the current distance measure of choice).

Finally, having calculated 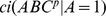 and 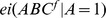, the total amount of *cause-effect information* (*cei*) specified by *A* = 1 over the purview 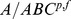 is the minimum of its *ci* and *ei*:
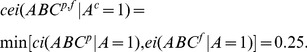
(3)


**Figure 5:**
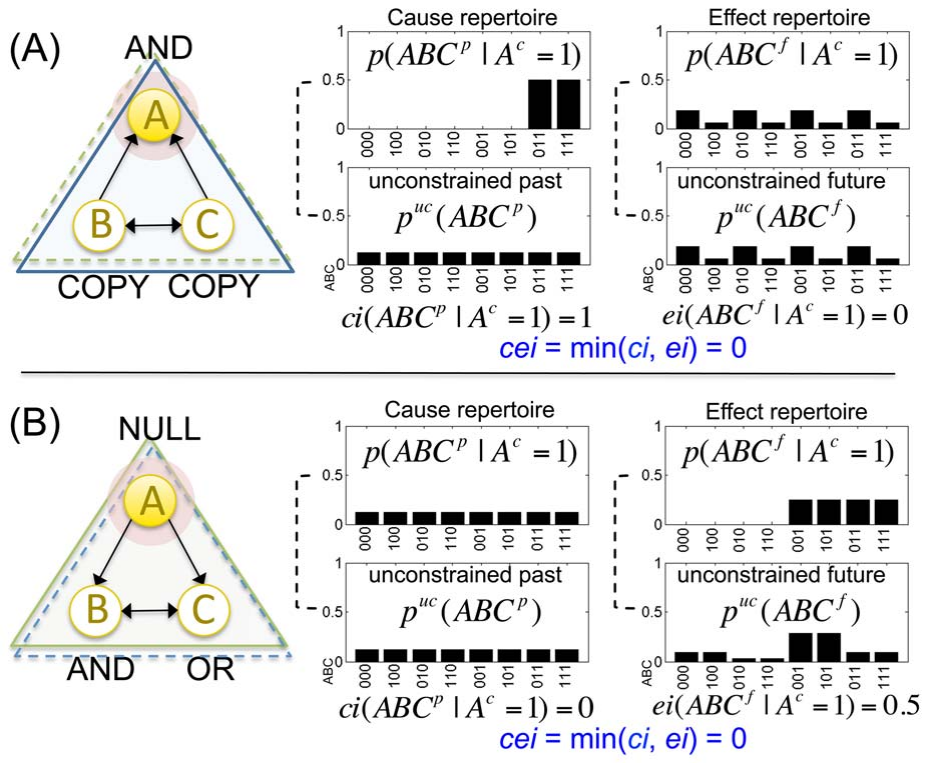
A mechanism generates information only if it has both selective causes and selective effects within the system. (A) Element *A* receives input from the system and specifies a selective cause repertoire. However, since it has no outputs to the system it does not specify a selective effect repertoire. (B) Element *A* receives no input from the system and therefore it does not specify a selective cause repertoire. In both cases the cause-effect information *cei* generated by mechanism *A* is zero (the minimum between cause and effect information).

The motivation for choosing the minimum is illustrated in Figure 5. First, consider an element that receives inputs from the system but sends no output to it (element *A* in Figure 5A). In this case, the state of element *A* constrains the past states of the system – *A* has selective causes within the system (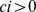), but not the future states of the system – *A* has no selective effects on the system (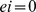, what *A* does makes no difference to the system). Put differently, while the state of element *A* does convey information about the system's past states from the perspective of an external observer, it does not do so from the intrinsic perspective of the system itself, because the system is not affected by *A* (the system cannot “observe” *A* and thus has no access to *A*'s cause information).

Similarly, consider an element that only outputs to the system but does not receive inputs from it, being controlled exclusively by external causes (element *A* in Figure 5B). In this case, the state of element *A* constrains the future states of the system – *A* has selective effects on the system (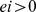), but not the past states of the system – *A* has no selective causes within the system (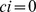, what the system might have done makes no difference to *A*). Put differently, while the state of element *A* does convey information about the system's future states from the perspective of an external observer, it does not do so from the intrinsic perspective of the system, because the system cannot affect the state of *A* (the system cannot “control” *A* and thus has no access to *A*'s effect information).

As illustrated by these two limiting cases, each mechanism in the system acts as an information bottleneck from the intrinsic perspective: its cause information only exists for the system to the extent that it also specifies effect information and vice versa. While other ways of measuring a mechanism's *cei* may also be compatible with the examples shown in Figure 5, the “intrinsic information bottleneck principle” is best captured by defining a mechanism's *cei* as the minimum between its cause and effect information.

#### Integration: Irreducible cause-effect repertoires and integrated information (*φ*)

**Figure 6:**
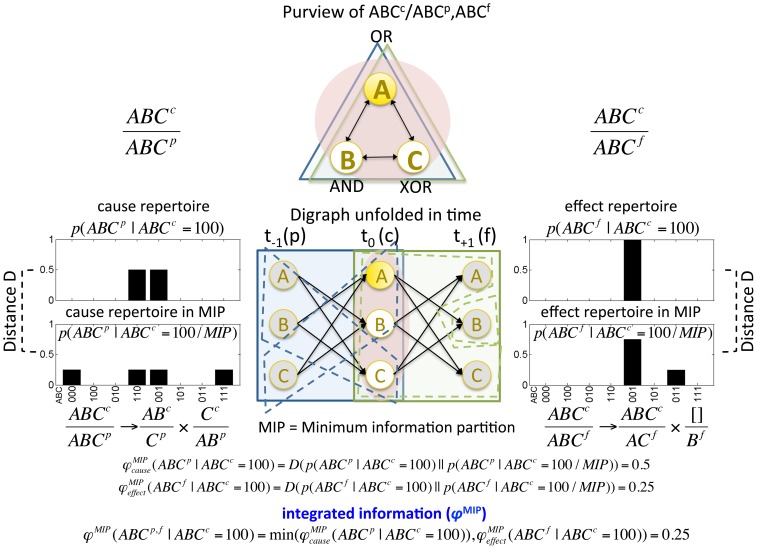
Integrated information: The information generated by the whole that is irreducible to the information generated by its parts. Integrated information is quantified by measuring the distance between the cause repertoire specified by the whole mechanism and the partitioned mechanism (the same for the effect repertoire). MIP is the minimum information partition – the partition of the mechanism that makes the least difference to the cause and effect repertoires (indicated by dashed lines in the unfolded system). Partitions are performed by noising connections between the parts (those that cross the dashed lines, see Text S2).

At the level of an individual mechanism, the integration postulate says that only mechanisms that specify integrated information can contribute to consciousness. Integrated information is information that is generated by the whole mechanism above and beyond the information generated by its parts. This means that, with respect to information, the mechanism is irreducible. Similar to cause-effect information, integrated information *φ* (“small phi”) is calculated as the distance *D* between two probability distributions: the cause-effect repertoire specified by the whole mechanism is compared against the cause-effect repertoire of the partitioned mechanism. Of the many possible ways to partition a mechanism, integrated information is evaluated across the minimum information partition (MIP), the partition that makes the least difference to the cause and effect repertoires (in other words, the minimum “difference” partition). In Figure 6 this is demonstrated for the 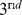 order mechanism *ABC*. The MIP for the purview 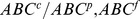 is 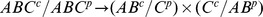 in the past and 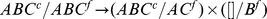 in the future, where [] denotes the empty set. The cause and effect repertoire specified by the partitioned mechanisms can be calculated as:
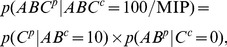
(4)and
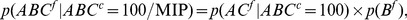
(5)where the connections between the parts are “injected” with independent noise (Text S2).

The distance *D* between the cause-effect repertoire specified by the whole mechanism and its MIP is quantified again using the EMD, taken separately for the past and the future (cause and effect repertoires):
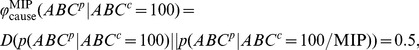
(6)

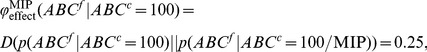
(7)


As with information, the total amount of integrated information of mechanism *ABC* in its current state 100 over the purview 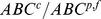 is the minimum of its past and future integrated information:
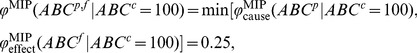
(8)In what follows, integrated information *φ* is always evaluated for the MIP, so the MIP superscript is dropped for readability.

**Figure 7:**
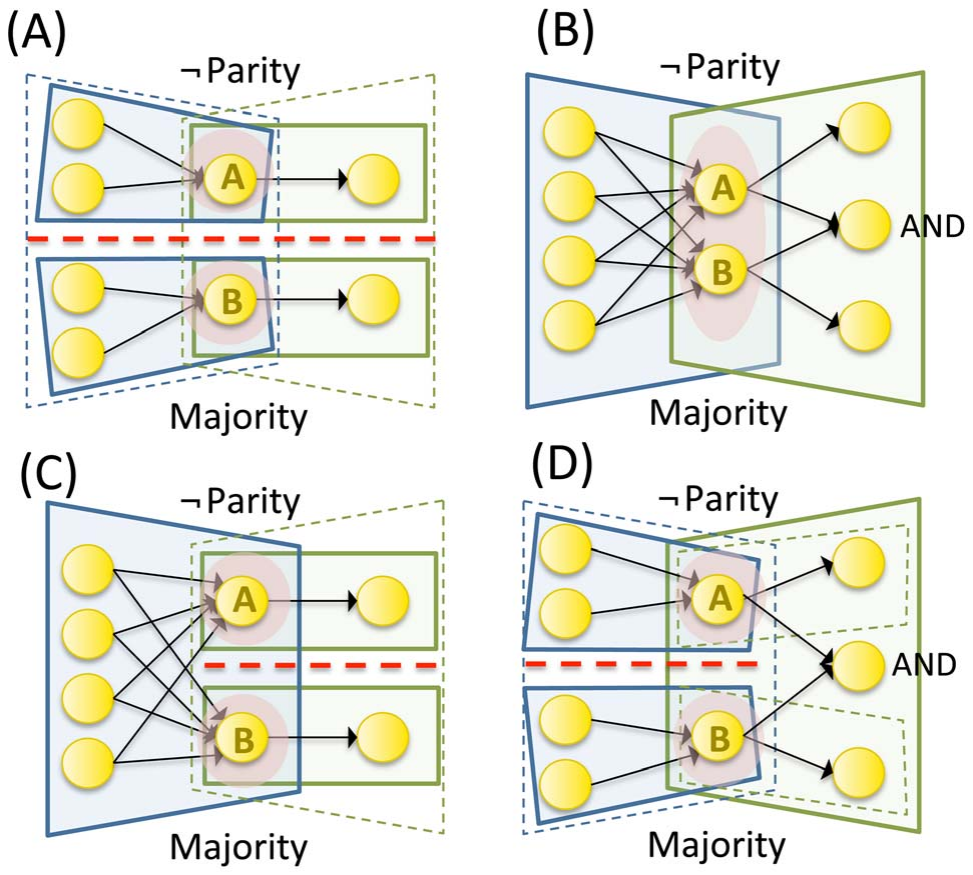
A mechanism generates integrated information only if it has both integrated causes and integrated effects. (A) The mechanisms of element *A* and *B* are independent, having separate causes and effects. From the intrinsic perspective of the system, the joint mechanism *AB* does not exist, since it can be partitioned (red dashed line) without making any difference to the system. (B) The mechanism *AB* generates integrated information both in the past and in the future. Since it cannot be partitioned without loss, it exists intrinsically. (C) The mechanism *AB* generates integrated information in the past but not in the future. (D) The mechanism *AB* generates integrated information in the future but not in the past. In both cases, the joint mechanism does not exist intrinsically.

According to IIT, mechanisms that do not generate integrated information do not exist from the intrinsic perspective of a system, as illustrated in Figure 7. Suppose that *A* is a non-parity gate (*A* turns on when the inputs are even) and *B* is a majority gate (*B* turns on when the majority of its inputs are on). If *A* and *B* have independent causes and independent effects as shown in Figure 7A, a higher order mechanism *AB* cannot generate integrated information, since it is possible to partition *AB*'s causes and effects without any loss of information. In this case, *AB* does not exist intrinsically.

Consider instead Figure 7B. Here, 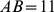 specifies that all inputs had to be on in the past (‘All ON’), which goes above and beyond what is specified separately by 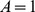 (an even number of inputs was on) and by 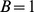 (the majority of inputs was on). On the effect side, there is an AND gate that takes inputs from both *A* and *B*, so the effect of 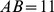 goes above and beyond the separate effects of 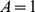 and 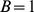. Therefore, mechanism *AB* exists from the intrinsic perspective of the system, in the sense that it plays an irreducible causal role: it picks up a difference that makes a difference to the system in a way that cannot be accounted for by its parts.

By contrast, in Figure 7C mechanism *AB* does not exist from the intrinsic perspective of the system, because the information ‘All ON’ as such does not make any difference to the future state of the system. Similarly, in Figure 7D, 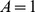 and 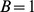 do not specify an irreducible past cause for the irreducible future effect that the AND gate will be ON.

#### Exclusion: A maximally irreducible cause-effect repertoire (MICE) specified by a subset of elements (a concept)

**Figure 8:**
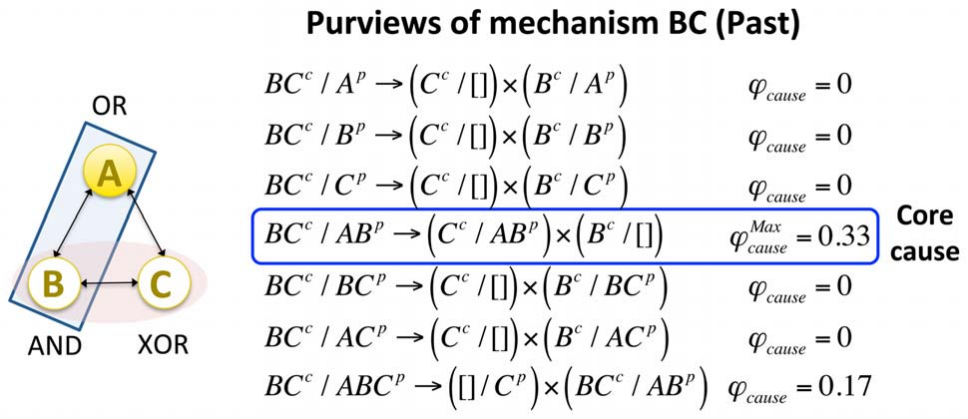
The maximally integrated cause repertoire over the power set of purviews is the “core cause” specified by a mechanism. All purviews of mechanism *BC* for the past are considered. Only the purview that generates the maximal value of integrated information, *φ* ^Max^, exists intrinsically as the core cause of the mechanism (or effect when considering the future). In this case, the core cause is 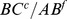.

The exclusion postulate at the level of a mechanism says that a mechanism can have only one cause and one effect, those that are maximally irreducible; other causes and effects are excluded. The *core cause* of a mechanism from the intrinsic perspective is its maximally irreducible cause repertoire (*one* cause thus means a probability distribution over the past states of *one* particular set of inputs of the mechanism). Consider for example mechanism 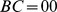 in Figure 8. To find the core cause of *BC*, one needs to evaluate 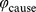 for all past purviews of the power set 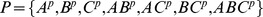. In this case, the purview 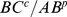 has the highest value of 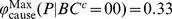. The corresponding maximally irreducible cause repertoire is thus the core cause of 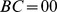. The *core effect* is assessed in the same way: it is the maximally irreducible effect repertoire of a mechanism with 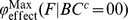, where *F* denotes the power set of future purviews. A mechanism that specifies a *maximally irreducible cause and effect (MICE)* constitutes a *concept* or, for emphasis, a *core concept*.

To understand the motivation behind the exclusion postulate as applied to a mechanism, consider a neuron with several strong synapses and many weak synapses (Figure S1). From the intrinsic perspective of the neuron, any combination of synapses could be a potential cause of firing, including “strong synapses”, “strong synapses plus some weak synapses”, and so on, eventually including the potential cause “all synapses”, “all synapses plus stray glutamate receptors”, “all synapses plus stray glutamate receptors plus cosmic rays affecting membrane channels”, and so on, rapidly escalating to infinite regress. The exclusion postulate requires, first, that only one cause exists. This requirement represents a causal version of Occam's razor, saying in essence that “causes should not be multiplied beyond necessity”, i.e. that causal superposition is not allowed [6]. In the present context this means that *only one* set of synapses can be the cause for the neuron's firing and not, for example, *both* “strong synapses S1,S2” *and* “all synapses”, or an average or integral over all possible causes. Second, the exclusion postulate requires that, from the intrinsic perspective of a mechanism in a system, the only cause be the maximally irreducible one. Recall that IIT's information postulate is based on the intuition that, for something to exist, it must make a difference. By extension, something exists all the more, the more of a difference it makes. The integration postulate further requires that, for a whole to exist, it must make a difference above and beyond its partition, i.e. it must be irreducible. Since, according to the exclusion postulate, only one cause can exist, it must be the cause that makes the most difference to the neuron's output if it is eliminated by a partition – that is, the cause that is maximally irreducible. In Figure S1, for example, the maximally irreducible cause turns out to be “the strong synapses S1,S2”. Note that the exclusion postulate appears to fit with phenomenology also at the level of mechanisms. Thus, invariant concepts such as “chair”, or “apple” seem to exclude the accidental details of particular apples and chairs, but only reflect the “core” concept. In neural terms, this would imply that the maximally irreducible cause-effect repertoire of the neurons underlying such invariant concepts is similarly restricted to their core causes and effects.

**Figure 9:**
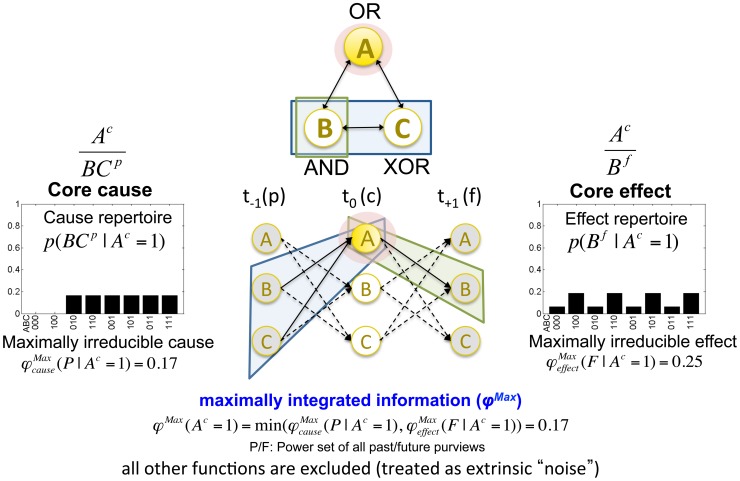
A concept: A mechanism that specifies a maximally irreducible cause-effect repertoire. The core cause and effect of mechanism *A* are 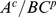 and 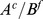, respectively. Together, they specify “what” the concept of *A* is about. The 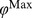 value of the concept specifies “how much” the concept exists intrinsically.

The notion of a concept is illustrated in Figure 9 for mechanism *A* of the candidate set *ABC*. The core cause of *A* is the cause repertoire of purview 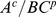; the core effect is the effect repertoire of 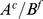. These purviews generate the maximal amount of integrated information over the whole power set of purviews in the past (*P*) and future (*F*), respectively. The amount of integrated information generated by concept 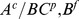 is again the minimum between past and future:
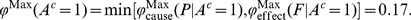
(9)Each concept of a mechanism in a state is thus endowed with a maximally irreducible cause-effect repertoire (MICE), which specifies what the concept is about (its *quale “sensu stricto”*), and its particular 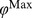 value, which quantifies its amount of integration or irreducibility. Finally note that the exclusion postulate is applied to the possible cause-effect repertoires of a single mechanism (elementary or higher order). Exclusion does not apply across mechanisms within a set of elements, since elementary and higher order mechanisms can have different causal roles (concepts) in the set, as emphasized by the composition postulate.

### Systems of mechanisms

We now turn from the level of mechanisms to the level of a system of mechanisms, and apply the postulates of IIT with the objective of deriving the experience or *quale* generated by a system in a bottom up manner, from the set of all its concepts.

#### Information: Conceptual structure (constellation of concepts in concept space) and conceptual information (*CI*)

**Figure 10:**
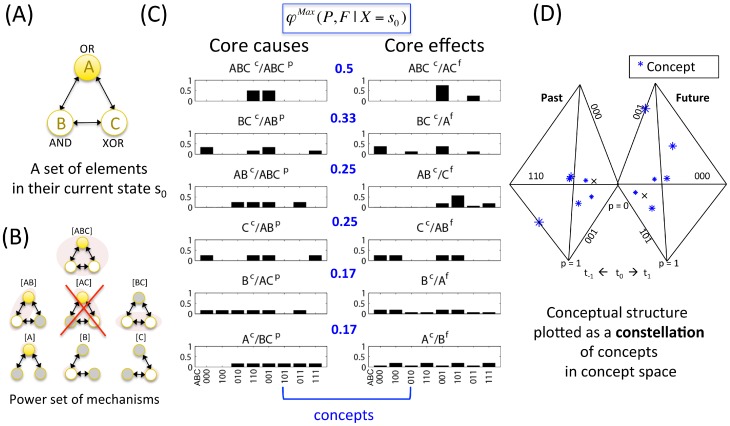
Information: A conceptual structure *C* (constellation of concepts) is the set of all concepts generated by a set of elements in a state. (A) The candidate set *ABC* – a system composed of mechanisms in a state. (B) The power set of *ABC*'s mechanisms. (C) The concepts generated by the candidate set. Core causes are plotted on the left, core effects on the right. 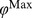 values are shown in blue fonts in the middle of the cause and effect repertoires of each mechanism. Note that all mechanisms in the power set are concepts, with the exception of mechanism *AC*, which can be fully reduced 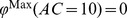. (D) The concepts generated by the candidate set plotted in concept space, where each axis corresponds to a possible state of *ABC*. For ease of representation past and future subspaces are plotted separately, with only three axes each. The “null” concept *p^uc^* is indicated by the small black crosses in concept space.

At the system level, the information postulate says that only sets of “differences that make a difference” (i.e. a constellations of concepts) matter for consciousness. Figure 10 shows all the concepts specified by the candidate set *ABC* (Figure 10A,B). Of all the possible mechanisms of the power set of *ABC*, only *AC* does not give rise to a concept, since its integrated information 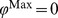 (Figure 10B). All other mechanisms generate non-zero integrated information and thus specify concepts (Figure 10C). The set of all concepts of a candidate set constitutes its *conceptual structure*, which can be represented in *concept space*.

Concept space is a high dimensional space, with one axis for each possible past and future state of the system. In this space, each concept is symbolized as a point, or “star”: its coordinates are given by the probability of past and future states in its cause-effect repertoire, and its size is given by its 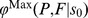 value. If 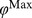 is zero, the concept simply does not exist, and if its 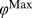 is small, it exists to a minimal amount.

In the case of the candidate set *ABC*, the dimension of concept space is 16 (8 axes for the past states and 8 for the future states). For ease of representation, in the figures past and future subspaces are plotted separately, with only three axes each (corresponding to the states at which the concepts have the highest variance in probability). Therefore the 6 concepts in Figure 10D are displayed twice, once in the past subspace and once in the future subspace. In the full 16-dimensional concept space, however, each concept is a single star.

**Figure 11:**
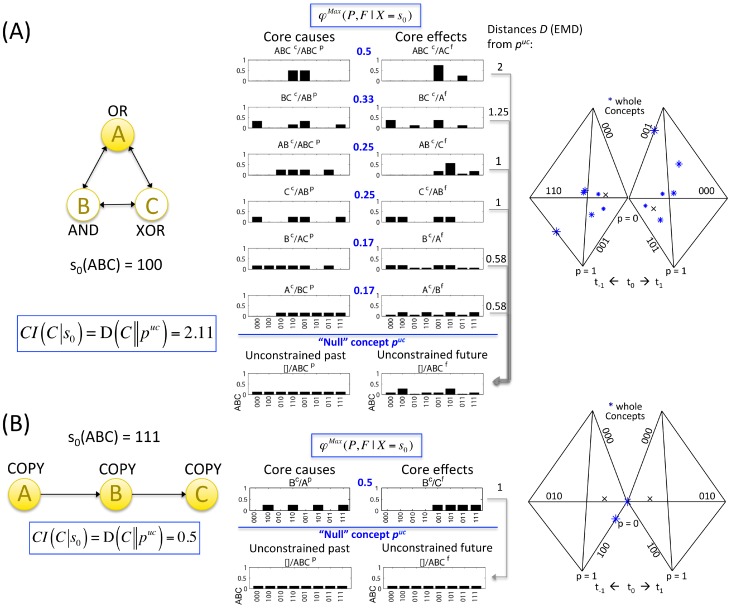
Assessing the conceptual information *CI* of a conceptual structure (constellation of concepts). *CI* is quantified by measuring the distance in concept space between *C*, the constellation of concepts generated by a set of elements, and 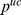, the unconstrained past and future repertoire, which can be termed the “null” concept (in the absence of a mechanism, every state is equally likely). This can be done using an extended version of the earth mover's distance (EMD) that corresponds to the sum of the standard EMD for distributions between the cause-effect repertoires of all concepts and 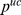, weighted by their 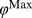 values. (A) Therefore, a system with many different elementary and higher order concepts has high *CI*, as shown here for the candidate set *ABC*. (B) By contrast, a system comprised of a single mechanism can only have one concept and thus has low *CI*.

At the system level, the equivalent of the cause-effect information (*cei*) at the level of mechanisms is called conceptual information (*CI*). Just like *cei*, *CI* is quantified by the distance *D* from the unconstrained repertoire of past and future states *p^uc^*, which corresponds to the “null” concept (a concept that specifies nothing):
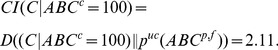
(10)The distance *D* from a constellation *C* to the “null” concept can be measured using an extension of the EMD (see Text S2), which can be understood as the cost of transporting the amount of 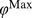 of each concept from its location in concept space to 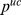. *CI* is thus the sum of the distances between the cause-effect repertoire of each concept and 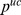, multiplied by the concept's 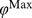 value (Figure 11). Thus, a rich constellation with many different elementary and higher order concepts generates a high amount of conceptual information *CI* (Figure 11A). By contrast, a system comprised of a single elementary mechanism generates a minimal amount of conceptual information (Figure 11B).

In sum, concepts are considered (metaphorically) as stars in concept space. The conceptual structure *C* generated by a set of mechanisms is thus a constellation of concepts – a particular shape in concept space spanned by the set's concepts. The more stars, the further away they are from the “null” concept, and the larger their size, the greater the conceptual information *CI* generated by the constellation *C*.

#### Integration: Irreducible conceptual structure and integrated conceptual information (Φ)

At the system level, the integration postulate says that only conceptual structures that are integrated can give rise to consciousness. As for mechanisms, the integration or irreducibility of the constellation of concepts *C* specified by a set of mechanisms can be assessed by partitioning a set of elements and measuring *integrated conceptual information* Φ as the difference made by the partition (“big phi”, as opposed to “small phi” *φ* at the level of mechanisms).

**Figure 12:**
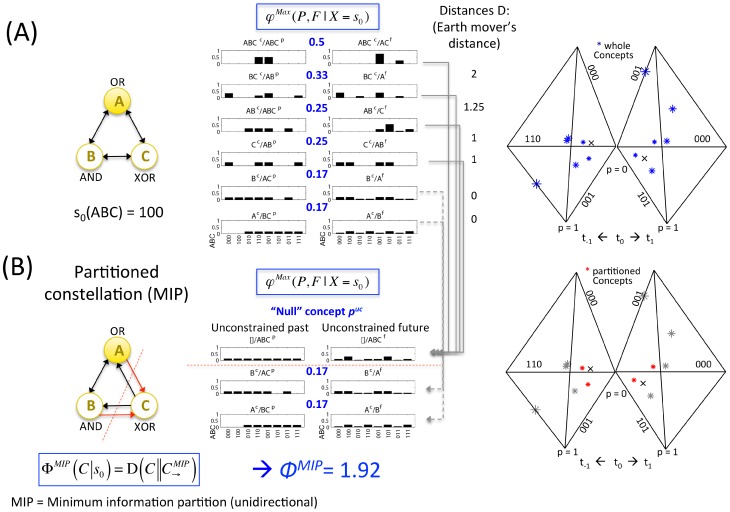
Assessing the integrated conceptual information Φ of a constellation *C*. Φ (“big phi”) is quantified by measuring the distance *C* between the constellation of concepts of the whole set of elements *C* and that of the partitioned set 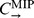, using an extended version of the earth mover's distance (EMD). The set is partitioned unidirectionally (see text for the motivation) until the partition is found that yields the least difference between the constellations (MIP, the minimum information i.e. minimum difference partition). In this case, the MIP corresponds to “noising” the connections from *AB* to *C*. This partition leaves 2 concepts intact (*A* and *B*, with zero distance to *A* and *B* from constellation *C*, indicated by the red stars), while the other concepts are destroyed by the partition (gray stars). The distance between the whole and partitioned constellations thus amounts to the sum of the EMD between the cause-effect repertoires of the destroyed concepts and the “null” concept 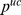, weighted by their 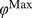 values (see Text S2).

Partitioning at the system level amounts to noising the connections from one subset 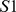 of *S* to its complement 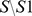. As for mechanisms, whether and how much the constellation of concepts generated by a set of mechanisms is irreducible can be assessed with respect to the minimum information partition (MIP) of the set of elements *S*. This corresponds to the unidirectional partition that makes the least difference to the constellation of concepts (in other words, the minimum “difference” partition; Figure 12). To find the unidirectional MIP, for each subset 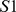 one must evaluate both the connections from 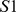 to 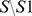 *and* the connections from 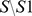 to 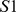 and take the minimum MIP. This corresponds, at the level of mechanisms, to finding the minimum of the MIPs with respect to the cause *and* the effect repertoires. Therefore a set of elements *S* and its associated constellation is integrated if and only if each subset of elements specifies both selective causes and selective effects about its complement in *S*. Similar to integrated information *φ* for a mechanism, integrated conceptual information Φ for a set of elements is defined as the distance *D* between the constellation of the whole set and that of the partitioned set:
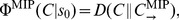
(11)where 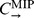 denotes the constellation of the unidirectionally partitioned set of elements.

The extended EMD between the whole and the partitioned constellation corresponds to the minimal cost of transforming *C* into 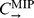 in concept space. Through the partition, concepts of *C* may change location, lose 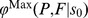, or disappear. Their 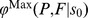 has to be allocated to fill the concepts in 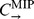 with an associated cost of transportation that is proportional to the distance in concept space and the amount of 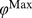 that is moved. Any residual 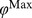 is transported to the “null” concept (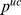) under the same cost of transportation.

Figure 12 shows the conceptual structure for the candidate system *ABC* and its MIP (see Text S2 for a calculation of 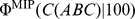). In this case, 4 of the 6 concepts of *ABC* are lost through the partition; their 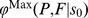 is thus transported to the location of the “null” concept (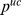). Since Φ is always evaluated over the MIP, in what follows the superscript MIP is dropped, as it was for *φ*.

**Figure 13:**
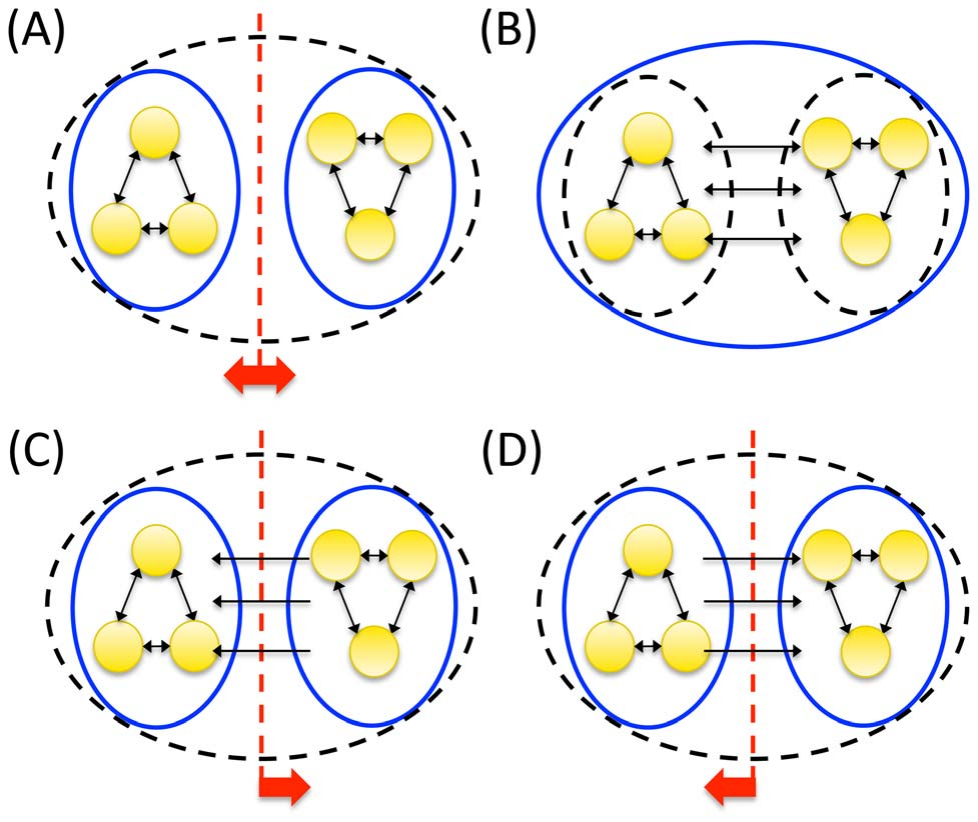
A set of elements generates integrated conceptual information Φ only if each subset has both causes and effects in the rest of the set. (A) A set of 6 elements is composed of two subsets that are not interconnected. The set reduces to 2 independent subsets of 3 elements each that can be partitioned without loss (dashed red line). The 6 element set does not exist intrinsically (dashed black oval). (B) All subsets of the 6 node set have causes and effects in the rest of the set. The 6 node set generates an integrated conceptual structure since it cannot be unidirectionally partitioned without loss of conceptual information. (C,D) A set of 6 elements divides into 2 subsets of 3 elements that are connected unidirectionally. (C) The left subset has causes in the rest of the set, but no effects. (D) The left subset has effects on the rest of the set, but no causes. In both cases, the set reduces to 2 subsystems of 3 elements each that can be unidirectionally partitioned without loss (dashed red line with directional arrow). The 6 element set does not exist intrinsically.

The motivation for integration at the system level is illustrated in Figure 13 (as was done for mechanisms in Figure 6). The set of 6 elements shown in Figure 13A can be subdivided into two independent subsets of 3 elements, each with its independent set of concepts. Therefore, a minimum partition between the two subsets makes no difference and integrated conceptual information 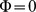. Since the set is reducible without any loss, it does not exist intrinsically – it can only be treated as “one” system from the extrinsic perspective of an observer. By contrast, the set in Figure 13B is irreducible because each part specifies both causes and effects in the other part. Two other possibilities are that a subset specifies causes, but not effects, in the rest of the set (Figure 13C), or only effects, but not causes (Figure 13D). In the case of unidirectional connections the subset is integrated “weakly” rather than “strongly” (in analogy with weak and strong connectedness in graph theory, e.g. [25]), which means that the subset is not really an “integral” part of the set, but merely an “appendix”. As an analogy, take the executive board of a company. An employee who transcribes the recording of a board meeting is obviously affected by the board, but if he has no way to provide any feed-back, he should not be considered an “integral” part of the board, which has no way of knowing that he exists and what he does. The same obtains for an employee who prints the agenda for the board meeting, if the board has no way of giving him feedback about the agenda.

#### Exclusion: A maximally irreducible conceptual structure (MICS) specified by a set of elements (a complex)

The exclusion postulate at the level of systems of mechanisms says that only a conceptual structure that is *maximally* irreducible can give rise to consciousness – other constellations generated by overlapping elements are excluded. A *complex* is thus defined as a set of elements within a system that generates a local maximum of integrated conceptual information Φ^Max^ (meaning that it has maximal Φ as compared to all overlapping sets of elements). Only a complex exists as an entity from the intrinsic perspective. Because of exclusion, complexes cannot overlap and at each point in time, an element/mechanism can belong to one complex only (complexes should be evaluated as maxima of integrated information not only over elements, but also over spatial and temporal grains [20], but here it is assumed that the binary elements and time intervals considered in the examples are optimal). Once a complex has been identified, concept space can be called “*qualia space*,” and the constellation of concepts can be called a “*quale ‘sensu lato’*”. A quale in the broad sense of the word is therefore a *maximally irreducible conceptual structure (MICS)* or, alternatively, an *integrated information structure*.

**Figure 14:**
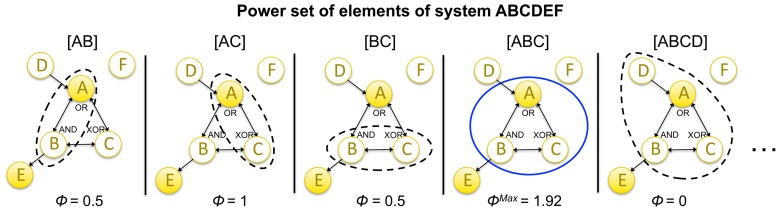
A complex: A local maximum of integrated conceptual information Φ. Integrated conceptual information Φ is computed for the power set of elements of system *ABCDEF* (all possible candidate sets). By the exclusion postulate, among overlapping candidate sets, only one set of elements forms a complex, the one that generates the maximum amount of integrated conceptual information Φ^Max^. In the example system the set of elements *ABC* form the complex. Therefore, no subset or superset of *ABC* can form another complex. Note that all candidate sets that include *D*, *E*, or *F* are not strongly integrated and thus have Φ = 0 (only one example is shown).

To determine whether an integrated set of elements is a complex, Φ must be evaluated for all possible candidate sets (subsets of the system) (Figure 14). As mentioned above, when a set of elements within the system is assessed, the other elements are treated as background conditions (see Text S2). Figure 14 shows the values of 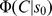 for all possible candidate sets that are subsets of 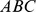 (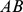,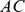,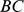,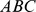) and for one superset (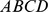). The latter, and all other sets that include elements *D*, *E*, or *F*, have Φ = 0. This is because *D*, *E*, and *F* are not strongly integrated with the rest of the system. Single elements are not taken into account as candidate sets since they cannot be partitioned and thus cannot be complexes by definition. In this example, the set of elements *ABC* generates the highest value of Φ^Max^ and is therefore the complex. By the exclusion postulate (“of all overlapping sets of elements, only one set can be conscious”), only *ABC* “exists” intrinsically, and other overlapping sets of elements within the system cannot “exist” intrinsically at the same time (they are excluded).

#### Identity between an experience and a maximally irreducible conceptual structure (MICS or quale “sensu lato”) generated by a complex

The notions and measures related to the information, integration, and exclusion postulates, both at the level of mechanisms and at the level of systems of mechanisms, are summarized in Table 1. On this basis, it is possible to formulate the central identity proposed by IIT: *an experience is identical with the maximally irreducible conceptual structure (MICS, integrated information structure, or quale “sensu lato”) specified by the mechanisms of a complex in a state*. Subsets of elements within the complex constitute the concepts that make up the MICS. The maximally irreducible cause-effect repertoire (MICE) of each concept specifies what the concept is about (what it contributes to the quality of the experience, i.e. its *quale “sensu stricto”* (in the narrow sense of the term)). The value of irreducibility *φ* ^Max^ of a concept specifies how much the concept is present in the experience. An experience (i.e. consciousness) is thus an *intrinsic property* of a complex of elements in a state: how they constrain – in a compositional manner – its space of possibilities, in the past and in the future.

**Figure 15:**
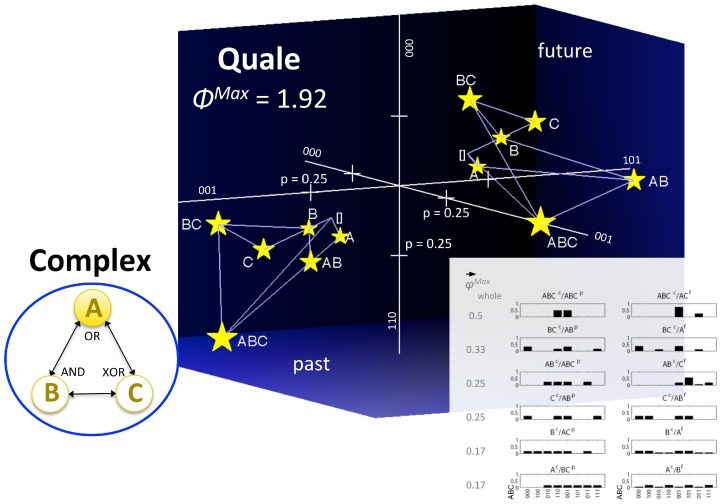
A quale: The maximally irreducible conceptual structure (MICS) generated by a complex. An experience is identical with the constellation of concepts specified by the mechanisms of the complex. The Φ^Max^ value of the complex corresponds to the quantity of the experience, the “shape” of the constellation of concepts in qualia space completely specifies the quality of a particular experience and distinguishes it from other experiences.

In Figure 15, this identity is illustrated by showing an isolated system of physical mechanisms ABC in a particular state (bottom left). The above analysis allows one to determine that in this case the system does constitute a complex, and that it specifies a MICS or quale (top right). As before, the constellation of concepts in qualia space is plotted over 3 representative axes separately for past and future states of the system. For clarity, the concepts are also represented as probability distributions over all 16 past and future states (cause-effect repertoires, bottom right).

The central identity of IIT can also be formulated to express the classic distinction between *level* and *content* of consciousness [26]: the quantity or level of consciousness corresponds to the Φ^Max^ value of the quale; the quality or content of the experience corresponds to the particular constellation of concepts that constitutes the quale – a particular shape in qualia space. Note that, by specifying the quality of an experience, the particular shape of each constellation also distinguishes it from other possible experiences, just like the particular shape of a tetrahedron is what makes it a tetrahedron and distinguishes it from a cube, an icosahedron, and so on.

s indicated by the figure, once a phenomenological analysis of the essential properties (axioms) of consciousness has been translated into a set of postulates that the physical mechanisms generating consciousness must satisfy, it becomes possible to invert the process: One can now ask, for any set of physical mechanisms, whether it is associated with phenomenology (is there “something it is like to be it,” from its own intrinsic perspective), how much of it (the quantity or level of consciousness), and of which kind (the quality or content of the experience). As also indicated by the figure, these phenomenological properties should be considered as intrinsic properties of physical mechanisms arranged in a certain way, meaning that a complex of physical mechanisms in a certain state is necessarily associated with its quale.

## Results/Discussion

The Models section presented a way of constructing the experience or quale generated by a system of mechanisms in a state in a step-by-step, bottom up manner. The next section explores several implications of the postulates and concepts introduced above using example systems of mechanisms and the conceptual structures they generate.

### A system may condense into a major complex and several minor complexes

**Figure 16:**
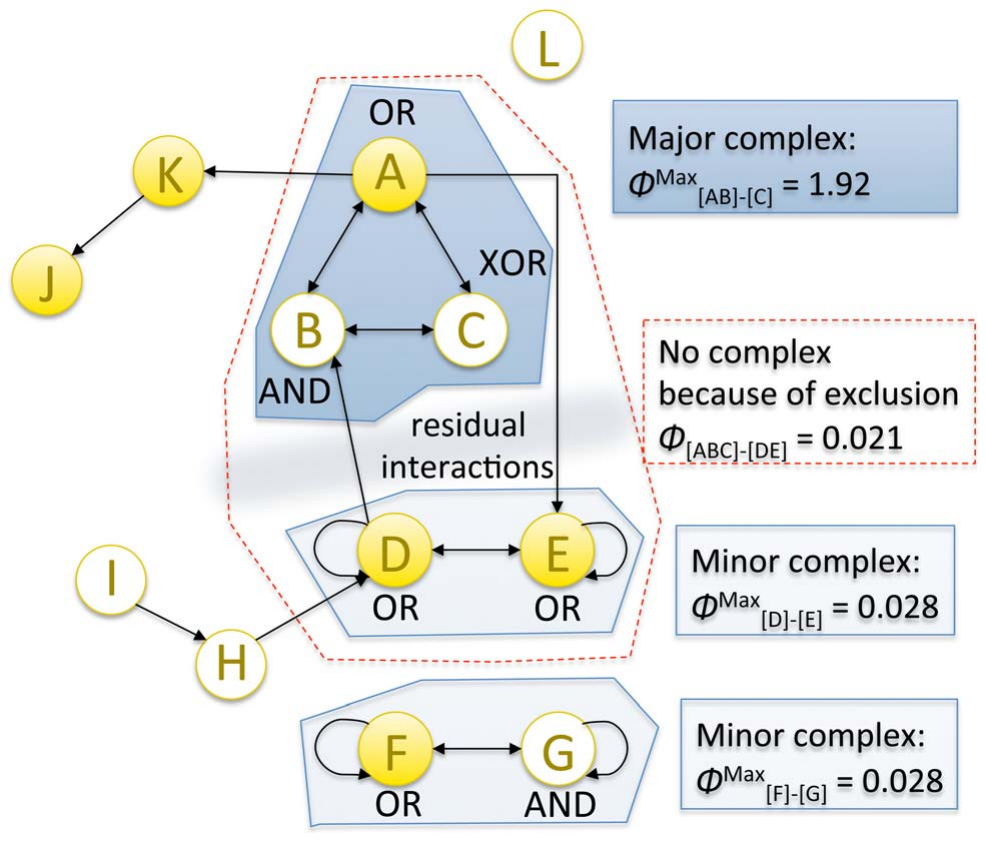
A system can condense into a major complex and minor complexes that may or may not interact with it. The set of elements *ABC* specifies the local maximum of integrated information Φ^Max^ and thus forms the major complex of the system. The sets of elements *DE* and *FG* also specify local maxima of integrated information albeit with lower Φ^Max^ than the main complex. *DE* and *FG* thus form minor complexes. The set of elements *ABCDE* is strongly integrated, but is excluded from forming a complex, since it overlaps with *ABC*, which is a local maximum of integrated information. The elements *I*, *J*, and *L* cannot be part of any complex since they do not have both causes and effects in the rest of the system. Neither can *H* and *K*, since they are part of a strictly feed-forward chain.

In Figure 16, the previous example system *ABC* has been embedded within a larger network. In the larger system, elements *I*, *J*, and *L* cannot be a part of the complex because they lack either inputs or outputs, or both. *H* and *K* also cannot be part of the complex, since they are connected to the rest of the system in a strictly feed-forward manner. Nevertheless, elements *H* and *K* act as background conditions for the rest of the system. The remaining elements 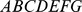 cannot form a complex as a whole, since the subset of elements *FG* is not connected to the rest of the system. The subset of elements 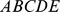 does generate a small amount of integrated conceptual information Φ and could thus potentially form a complex. Among the power set of elements 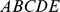, however, it is the smaller subset 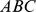 that generates the local maximum of Φ^Max^. This excludes 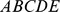 from being a complex, since an element can participate in only one complex at each point in time. The remaining elements *DE*, however, can still form a *minor complex*, with lower Φ^Max^ than *ABC*. Thus, 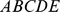 condenses down to the major complex *ABC*, the minor complex *DE*, and their residual interactions. Finally, *FG* forms a minor complex that does not interact with the rest of the system.

This simple example of “condensation” into major and minor complexes may be relevant also for much more complicated systems of interconnected elements. For example, IIT predicts that in the human brain there should be a dominant “main” complex of high Φ^Max^, constituted of neural elements within the cortical system, which satisfies the postulates described above and generates the changing qualia of waking consciousness [12]. The set of neuronal elements constituting this main complex is likely to be dynamic [27], at times including and at times excluding particular subsets of neurons. Through its interface elements (called “ports-in” and “ports-out”), this main complex receives inputs and provides outputs to a vast number of smaller systems involved in parsing inputs and planning and executing outputs. While interacting with the main complex in both directions, many of these smaller systems may constitute minor complexes specifying little more than a few concepts, which would qualify them as “minimally conscious” (see below). In the healthy, adult human brain the qualia and Φ^Max^ generated by the dominant main complex are likely to dwarf those specified by the minimally conscious minor complexes. In addition to the fully conscious main complex and minimally conscious minor complexes, there will be a multitude of unconscious processes mediated by purely feed-forward systems (see below) or by the residual interactions between main complex and minor complexes, as in Figure 16.

Under special circumstances, such as after split brain surgery, the main complex may split into two main complexes, both having high Φ^Max^. There is solid evidence that in such cases consciousness itself splits in two individual consciousnesses that are unaware of each other [28]. A similar situation may occur in dissociative and conversion disorders, where splits of the main complex may be functional and reversible rather than structural and permanent [29].

An intriguing dilemma is posed by behaviors that would seem to require a substantial amount of cognitive integration, such as semantic judgments (e.g. [30], [31]). Such behaviors are usually assumed to be mediated by neural systems that are unconscious, because they can be shown to occur under experimental conditions, such as continuous flash suppression, where the speaking subject is not aware of them and cannot report about them. If such behaviors were carried out in a purely feed-forward manner, they would indeed qualify as unconscious in IIT (see below). However, at least some of these behaviors may constitute the output of minor complexes separated from the main one. According to IIT such minor complexes, if endowed with non-trivial values of Φ^Max^, should be considered *paraconscious* (i.e. conscious “on the side” of the conscious subject) rather than unconscious. In principle, the presence of paraconscious minor complexes could be demonstrated by developing experimental paradigms of dual report.

In brains substantially different from ours many other scenarios may occur. For example, the nervous system of highly intelligent invertebrates such as the octopus contains a central brain as well as large populations of neurons distributed in the nerve cords of its arms. It is an open question whether such a brain would give rise to a large, distributed main complex, or to multiple major complexes that generate separate consciousnesses. Similar issues apply to systems composed of non-neural elements, such as ant colonies, computer architectures, and so on. While determining rigorously how such systems condense in terms of major and minor complexes, and what kind of MICS they may generate, is not practically feasible, the predictions of IIT are in principle testable and should lead to definite answers.

### Consciousness and connectivity: Modular, homogeneous, and specialized networks

**Figure 17:**
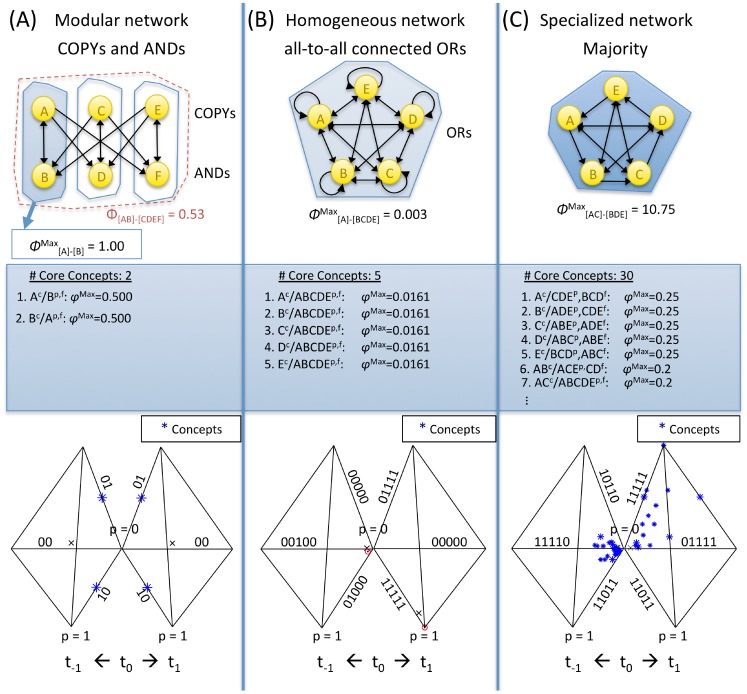
Qualia generated by modular, homogeneous and specialized networks. (A) The modular network decomposes into three small complexes and their residual interactions. (B) The homogenous system forms a complex, but it has low Φ^Max^ and only 5 identical concepts. (C) The specialized network also forms a complex, with all but one concepts of its power set and a high Φ^Max^ value. In the middle row, the respective concepts of each system are listed. The bottom row shows the constellation of the respective complexes in qualia space (projected into 3 dimensions for the past and the future subspaces).

Whether a set of elements as a whole constitutes a complex or decomposes into several complexes depends first of all on the connectivity among its elementary mechanisms. In Figure 17 we show the complexes and the associated MICS of three simple networks, representative of a modular, homogeneous, and specialized system architecture.

Figure 17A (top) shows a “modular” network of 3 COPY (*ACE*) and 3 AND (*BDF*) logic gates. In this network, the system as a whole is not a complex, despite being integrated due to the presence of inter-connections among all elements. Instead, each of the three modules (*AB*, *CD*, and *EF*) that consist of 1 COPY and 1 AND gate constitutes a complex, because each generates more Φ than the whole system, although each module has just two concepts. The purviews of module *AB*'s concepts are shown in Figure 17A (middle), and their representation in qualia space is displayed in Figure 17A (bottom).

Figure 17B shows a “homogeneous” network of 5 OR gates (*ABCDE*), in which every element is connected to every other element including itself. Since all elements in the network specify the same cause-effect repertoire, their 5 first order (elementary) concepts are identical. Moreover, there are no higher order concepts, since combining elements yields nothing above the elementary mechanisms. In qualia space, the 5 identical concepts are concentrated on a single point (Figure 17B, bottom). Accordingly, the homogeneous network has a low value of *CI* and Φ^Max^.

Figure 17C shows a “specialized” network consisting of 5 majority gates, which turn on when the majority of inputs is on. However, each gate has only 3 afferent and efferent connections, which differ for every element. Therefore, each elementary concept specifies a different cause-effect repertoire. For the same reason, there are many higher order concepts (all but the highest order concept of the power set). The specialized network thus gives rise to a rich constellation in qualia space (Figure 17C, bottom) with a high value of *CI* and Φ^Max^.

The example in Figure 17A, which shows that a network can be interconnected, either directly or indirectly, yet condense into a number of mini-complexes of low Φ^Max^ if its architecture is primarily modular, is potentially consistent with neuropsychological evidence. As mentioned in the Introduction, the cerebellum is a paramount example of a complicated neuronal network, comprising even more neurons than the cerebral cortex, that does not give rise to consciousness or contribute to it [32]–[34]. This paradox could be explained by its anatomical and physiological organization, which seems to be such that small cerebellar modules process inputs and produce outputs largely independent of each other [35], [36]. By contrast, a prominent feature of the cerebral cortex, which instead can generate consciousness, is that it is comprised of elements that are functionally specialized and at the same time can interact rapidly and effectively [4], [37], [38]. This is the kind of organization that yields a comparatively high value of Φ^Max^ in the simple example of Figure 17C. Finally, the example in Figure 17B, where connections are abundant but are organized in a homogeneous manner, may also have neurobiological counterparts. For instance, during deep slow wave sleep or in certain states of general anesthesia, the interactions among different cortical regions become highly stereotypical. Due to the characteristic bistability between on and off states of most neurons in the cerebral cortex, even though the anatomical connectivity is unchanged, functional and effective connectivity become virtually homogeneous [39], [40]. Under such conditions, consciousness invariably fades [14]. The examples of Figure 17B and C also suggest that both the richness of concepts and the level of consciousness should increase with the refinement of cortical connections during neural development and the associate increase in functional specialization (e.g. [41]).

### Consciousness and activity: Inactive systems can be conscious

The conceptual structure generated by a complex depends not only on the connectivity among its elements and the input/output function they perform, but also on their current state. An important corollary of IIT is that both active and inactive elements can contribute to its conceptual structure. Moreover, high-order concepts will often be specified by subsets including both active and inactive elements.

**Figure 18:**
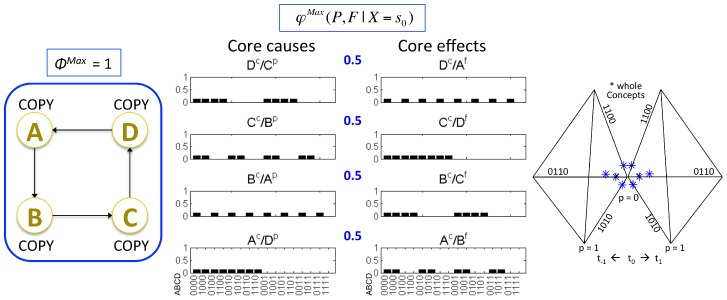
Quale generated by an inactive system. Neural activity is not necessary to generate experience, nor does it need to be “broadcasted” globally. Although all the elements in the system are off (0), the system still forms a complex and specifies a MICS. Moreover, an element can contribute to experience as long as it affects the shape of the MICS, without the need to “broadcast” its activity globally to affect every other element. This is because information is not in the message that is broadcasted by an element, but it is the shape of the MICS that is specified by a complex.

In Figure 18, the system ABCD, comprised of 4 COPY gates, illustrates that a set of elements can form a complex and specify a MICS even though *all* of its elements are in state ‘0’ (off). This is because inactive elements, too, can selectively constrain past and future states of the system (as opposed to “inactivated” or non-functional elements, which cannot change state and thus cannot generate information). For example, element 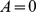 specifies an irreducible cause (*D* had to be off at 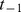) and an irreducible effect (*B* will be on at 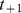) within the complex. Thus, IIT predicts that, even if all the neurons in a main complex were inactive (or active at a low baseline rate), they would still generate consciousness as long as they are ready to respond to incoming spikes. An intriguing possibility is that a neurophysiological state of near-silence may be approximated through certain meditative practices that aim at reaching a state of “pure” awareness without content [18], [42]. This corollary of IIT contrasts with the common assumption that neurons can only contribute to consciousness if they are active in such a way that they can “signal” or “broadcast” the information they represent and “ignite” fronto-parietal networks [7], [10]. This is because, in IIT, information is not in the message that is broadcasted by an element, but in the shape of the MICS that is specified by a complex.

Another corollary of IIT that is relevant to neuroscience is that it is not necessary for the firing state of neurons to percolate or be “broadcasted” globally through the entire main complex for it to contribute to experience. For example, in the system in Figure 18, element *A* does not connect directly to element *C*. As a consequence, the activity (or inactivity) of *A* cannot affect *C*, and vice versa, within one time step. Nevertheless, *ABCD* still forms a complex and gives rise to a MICS at time 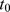. Thus, according to IIT, the activation or deactivation of a neuron (over the time scale at which integrated information reaches a maximum [20]) can modify an experience as long as it affects the shape of the MICS specified by the complex to which the neuron belongs, without requiring any global “broadcast” of signals.

### Simple systems can be conscious: A “minimally conscious” photodiode

The previous section showed that activations and direct interactions between elements are not necessary to generate a MICS. Taking into account the axioms and postulates of IIT, we can now summarize what it takes to be conscious and give an example of a “minimally conscious system,” which will be called a “minimally conscious” photodiode.

**Figure 19:**
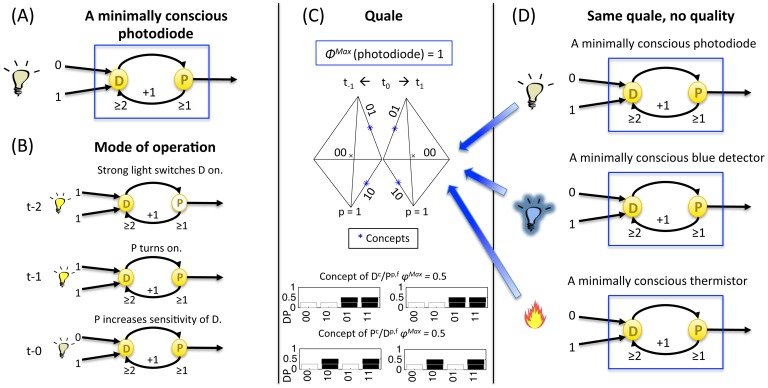
Quantity and quality of experience of a “minimally conscious” photodiode. (A) The minimally conscious photodiode *DP* consists of detector element *D* and predictor element *P*. *D* receives two external inputs and has a threshold ≥2. All connections have weight 1. (B) *P* serves as a memory for the previous state of *D* and its feed-back to *D* serves as a predictor of the next external input by effectively decreasing the threshold of *D*. (C) The MICS specified by the minimally conscious photodiode. *D* and *P* both specify a first order concept about the other element. (D) A minimally conscious thermistor or a minimally conscious blue detector with the same internal mechanisms as the minimally conscious photodiode generate the same MICS and therefore have the same minimal experience.

The “photodiode” in Figure 19A consists of two elements: the detector *D* and the predictor *P*. *D* receives two external light inputs (and is thus a port-in) and one internal input from *P*, all with strength 1. As illustrated in Figure 19B, *D* turns on if it receives at least two inputs from internal and/or external sources. If *D* has switched on due to sufficiently strong external inputs, it activates element *P*, which serves as a “memory”. At the next time step, *P* acts as a “predictor” of the next external input to *D* by increasing its sensitivity to light.

Simple as it is, the photodiode system satisfies the postulates of IIT: both of its elements specify selective causes and effects within the system (each element about the other one), their cause-effect repertoires are maximally irreducible, and the conceptual structure specified by the two elements is also maximally irreducible. Consequently, the system 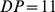 forms a complex that gives rise to a MICS, albeit one having just two concepts and a Φ^Max^ value of 1 (Figure 19C). *DP* is therefore conscious, albeit minimally so.

It is instructive to consider the quality of experience specified by such a minimally conscious photodiode. From an observer's perspective, the photodiode detects light, but from the intrinsic perspective, the experience is only minimally specified, and in no way can convey the meaning “light”: *D* says something about *P*'s past and future, and *P* about *D*'s, and that is all. Accordingly, the shape in qualia space is a constellation having just two stars, and is thus minimally specific. This aspect is further emphasized if one considers that different physical systems, say a photodiode activated by blue light (a “blue” detector), or even a binary thermistor (a “temperature” detector) would generate the exact same MICS (Figure 19D) and thus the same minimal experience. Moreover, the symmetry of the MICS implies that the quality of the experience would be the same regardless of the system's state: the photodiode in state 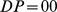, 01, or 10, receiving one external input, generates exactly the same MICS as 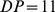. In all the above cases, the experience might be described roughly as “it is like this rather than not like this”, with no further qualifications. The photodiode's experience is thus both quantitatively and qualitatively minimal. Only additional mechanisms that create new concepts and break the symmetries in the shape of the MICS can generate additional meaning. Ultimately, only a set of concepts comparable to that of our main complex can specify the shape of the experience “light” as it appears to us, and distinguish it from countless other shapes corresponding to different experiences [6].

### Complex systems can be unconscious: A “zombie” feed-forward network

**Figure 20:**
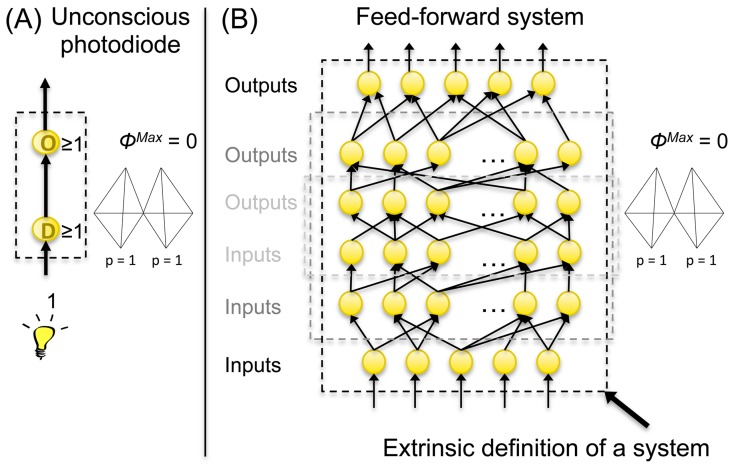
Feed-forward “zombie” systems do not generate consciousness. (A) An unconscious photodiode *DO* without recurrent connections. The detector element *D* affects output element *O*, but has no cause within the system *DO*. *O* is caused by *D*, but has no effect on the photodiode *DO*. Therefore, the elements do not form a complex and generate no quale. (B) Even complicated systems cannot form a complex if they have a strictly feed-forward architecture. This can be understood in the following way: for any system background imposed by an observer, the system's input layer has no causes within the system and the output layer has no effects on it, regardless of the elements' (logic) functions. Consequently, the system cannot form a complex and it remains unconscious, just like the unconscious photodiode *DO*.

Another corollary of IIT is that certain structures do not give rise to consciousness even though they may perform complicated functions. Consider first an “unconscious” photodiode (Figure 20A), comprising again two elements: a detector *D* and output *O*. In this case, however, whether *D* is on or off is determined by external inputs only, and the output of *O* does not feed back into the system. Therefore, *D*'s response to light is just passed through the system, but never comes back to it. Although an observer may describe the two elements *DO* as a system, *D* and *O* do not have both causes and effects within the system *DO*, which is thus not a complex, and generates no quale.

The same lack of feed-back that disqualifies the unconscious photodiode can be extended, by recursion, to any feed-forward system, no matter how numerous its elements and complicated its connectivity (Figure 20B). From the viewpoint of an extrinsic observer, the system's borders can be set arbitrarily. However, the input layer is always determined entirely by external inputs and the output layer does not affect the rest of the system. Consequently, from the intrinsic perspective, both input and output layer cannot be part of the complex. Drawing the system boundaries closer and closer together in a recursive manner, one eventually ends up with just one input and output layer, made up of many “unconscious photodiodes”, and thus generating no quale. Therefore, systems with a purely feed-forward architecture cannot generate consciousness.

The idea that “feed-back”, “reentry”, or “recursion” of some kind may be an essential ingredient of consciousness has many proponents [27], [43]–[45]. Recently, it has been suggested that the presence or absence of feed-back could be directly equated with the presence or absence of consciousness [46]. Moreover, several recent studies indicate that an impairment of reentrant interactions over feed-back connections is associated with loss of consciousness during anesthesia [47]–[49] and in brain-damaged patients [50]. However, it has been pointed out that the brain (and many other systems) is full of reentrant circuits, many of which do not seem to contribute to consciousness [51]. IIT offers some specific insights with respect to these issues. First, the need for reciprocal interactions within a complex is not merely an empirical observation, but it has theoretical validity because it is derived directly from the phenomenological axiom of (strong) integration. Second, (strong) integration is by no means the only requirement for consciousness, but must be complemented by information and exclusion. Third, for IIT it is the potential for interactions among the parts of a complex that matters and not the actual occurrence of “feed-back” or “reentrant” signaling, as is usually assumed. As was discussed above, a complex can be conscious, at least in principle, even though none of its neurons may be firing, no feed-back or reentrant loop may be activated, and no “ignition” may have occurred.

### Conscious complexes and unconscious “zombie” systems can be functionally equivalent

The last section showed that according to IIT feed-forward systems cannot give rise to a quale. However, without restrictions on the number of nodes, feed-forward networks with multiple layers can in principle approximate almost any given function to an arbitrary (but finite) degree [52], [53]. Therefore, it is conceivable that an unconscious system could show the same input-output behavior as a “conscious” system.

**Figure 21:**
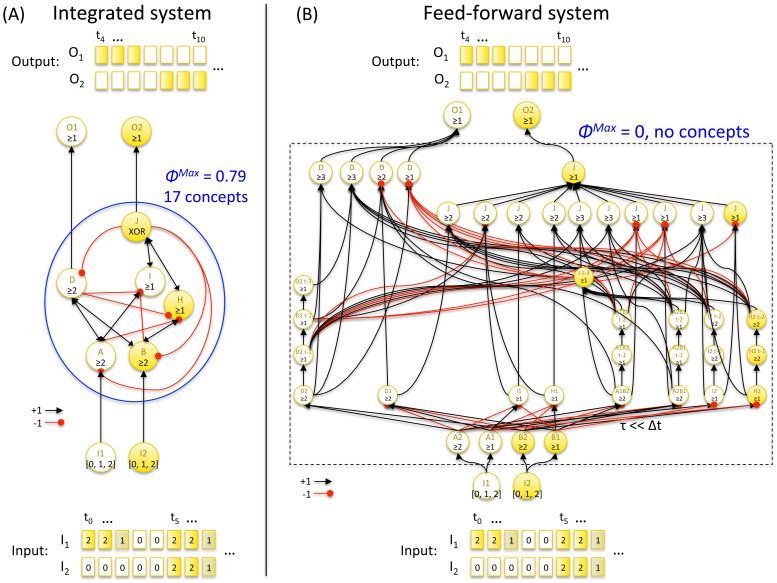
Functionally equivalent conscious and unconscious systems. (A) A strongly integrated system gives rise to a complex in every network state. In the depicted state (yellow: 1, white: 0), elements 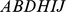 form a complex with Φ^Max^ = 0.76 and 17 concepts. (B) Given many more elements and connections, it is possible to construct a feed-forward network implementing the same input-output function as the strongly integrated system in (A) for a certain number of time steps (here at least 4). This is done by unfolding the elements over time, keeping the memory of their past state in a feed-forward chain. The transition from the first layer to the second hidden layer in the feed-forward system is assumed to be faster than in the integrated system (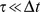) to compensate for the additional layers (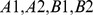). Despite the functional equivalence, the feed-forward system is unconscious, a “zombie” without phenomenological experience, since its elements do not form a complex.

An example is shown in Figure 21A. A strongly integrated system is compared to a feed-forward network that produces the same input-output behavior over at least 4 time steps (9^4^ input states, Figure 21B). To achieve a memory of *x* past time steps in the feed-forward system, the relevant elements were unfolded over time: the state of each element is passed on through a chain of *x* nodes, one node for each of the *x* time steps [54], [55]. In this way, the states of upstream elements in previous time steps can be combined (converge) in a feed-forward manner to determine the state of elements downstream, but can never feed back on elements upstream. As illustrated in the figure, while the recurrent system gives rise to a complex with Φ^Max^>0 in every state, and would therefore be conscious, the feed-forward system does not constitute a complex and is thus unconscious.

This comparison highlights an important corollary of IIT: whether a system is conscious or not cannot be decided based on its input-output behavior only. In neuroscience, the ability to report is usually considered as the gold standard for assessing the presence of consciousness. Behavior and reportability can be reliable guides under ordinary conditions (typically adult awake humans) and can be employed to evaluate neural correlates of consciousness [9] and to validate theoretical constructs [14]. However, behavior and reportability become problematic for evaluating consciousness in pathological conditions, during development, in animals very different from us, and in machines that may perform sophisticated behaviors [6]. For example, programs running on powerful computers can not only play chess better than humans, but win in difficult question games such as “Jeopardy” [3]. Moreover, recent advances in machine learning have made it possible to construct simulated networks, primarily feed-forward, that can learn to recognize natural categories such as cats, dogs [1], pedestrians [56], [57], and/or faces [58]–[60]. Hence, if behavior is the gold standard, it is not clear on what grounds we should deny consciousness to a phone “assistant” program that can answer many difficult questions, and can even be made to report about her internal feelings, or to a chip that recognizes thousands of different objects as well or better than we do, while granting it to a human who can barely follow an object with his eyes. IIT claims, by contrast, that input-output behavior is not always a reliable guide: one needs to investigate not just “what” functions are being performed by a system, but also “how” they are performed within the system. Thus, IIT admits the possibility of true “zombies”, which may behave more and more like us while lacking subjective experience [11].

The examples of Figure 21 also suggest that, while it may be possible to build unconscious systems that perform many complex functions, there is an evident evolutionary advantage towards the selection of integrated architectures that can perform the same functions consciously. Among the benefits of integrated architectures are economy of units and wiring, speed, compositionality, context-dependency, memory, and the ability to learn adaptive functions rapidly, flexibly, and building upon previous knowledge [6]. Moreover, in a feed-forward network all system elements are entirely determined by the momentary external input passing through the system. By contrast, a (strongly) integrated system is autonomous, since it can act and react based on its internal states and goals.

### The concepts within a complex are self-generated, self-referential, and holistic

**Figure 22:**
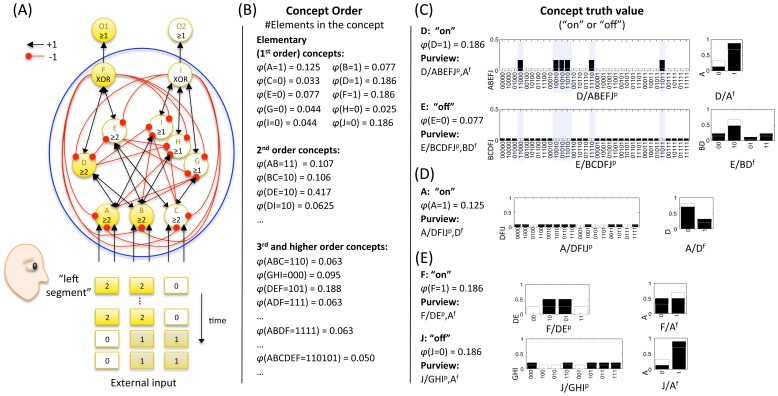
A complex can have ports-in and ports-out from and to the external environment, but its qualia are solipsistic: Self-generated, self-referential, and holistic. (A) A recurrent segment/dot system consisting of 10 elements (8 linear threshold units, and 2 XOR logic gates) that are linked by excitatory and inhibitory connections (black +1, red −1). 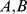 and *C* are the ports-in of the complex. They receive external inputs of strength 0, 1, or 2. Elements *F* and *J* are the ports-out of the complex. They output to the external elements *O*1 and *O*2. The current state of the system corresponds to a sustained input with value 2-2-0. From an extrinsic perspective, the different layers of the complex can be interpreted as feature detectors having increasingly invariant selectivities (e.g. *D* indicates “two contiguous left elements”, *F* “invariant segment”, and *J* “invariant dot”). (B) Since the segment/dot system is highly interconnected with specialized mechanisms, all first order concepts and many higher order concepts exist. (C) Both, elementary mechanisms that are “on” (1) and those that are “off” (0) constitute concepts. Note that the cause repertoire of 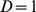 is the mirror image of the cause repertoire of 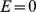 (highlighted in blue). (C,D,E) From the intrinsic perspective, the function of a mechanism is given by its cause-effect repertoire. The purview of a concept can only contain elements within the complex. The concepts that constitute the MICS generated by the complex are self-generated (specified exclusively by elements belonging to the complex); self-referential (specified exclusively over elements belonging to the complex); and holistic (their meaning is constructed in the context of the other concepts in the MICS).

The final example (Figure 22A) considers a simple perceptual system – a recurrent segment/dot system. The segment/dot system consists of 10 heavily interconnected elements that, in their current state, form a complex (Figure 22A, blue circle). Elements 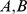, and *C* are the ports-in of the complex: they each receive 2 inputs from an external source in addition to feed-back inputs from within the complex. Elements *F* and *J* are the ports-out of the complex: they output to the external elements *O*1 and *O*2, respectively, in addition to their outputs within the complex. In this example, the ports-out are XOR logic gates. All other elements inside the segment/dot system are linear threshold units (LTUs). Connections within the complex are excitatory (+1, black) or inhibitory (−1, red).

The elementary mechanisms comprising the segment/dot system have specialized functions and generate elementary concepts. In the segment/dot system, the concepts of mechanisms in the “off” state (0) tend to have lower *φ* ^Max^ values, because the mechanisms tend to be more selective in their “on” state (1) (see also Figure 3). As listed in Figure 22B, in addition to first order concepts, the segment-dot system gives rise to many higher order concepts. Dependent on the state of the system, certain higher order concepts may or may not exist. For instance, in the current state of the segment/dot system, the second order concept *DI* exists, while *EG* does not because it is reducible (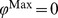). If the segment/dot system were presented instead with a “right”-segment (inputs 022), *DI* would disappear and *EG* would emerge.

From the perspective of an external observer (e.g. a neuroscientist recording the activity of “neurons” 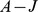), the function of a mechanism is typically described with respect to external inputs (e.g. a “segment” detector). In the segment/dot system, mechanisms at different hierarchical levels correspond to increasing levels of invariance: element *D*, for example, turns on if the two contiguous pixels on the left have been on persistently (with inputs of strength 2); higher up in the system, element *F* turns on if two contiguous pixels have been on either on the left or on the right, thus indicating the presence of the invariant “segment”. Element *J*, on the other hand, detects the invariant “dot”, either left, right, or center. The excitatory and inhibitory feed-back connections in the segment/dot system serve a predictive function: they temporarily increase/decrease the sensitivity to similar/opposed stimuli, allowing weaker inputs (with a value of 1) to be detected as segments and dots if the weaker external input is in accordance with the feed-back from within the complex.

From the intrinsic perspective of the system, instead, the function of each mechanism is given by its concept. Each concept is *self-generated*, because it must be specified exclusively by a subset of elements belonging to the complex. It is also *self-referential*, because its cause-effect repertoire refers exclusively to elements within the complex, and therefore only indirectly to external inputs. For example, the concept of *D*, in its current state 1, is about the purview 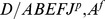. From the intrinsic perspective, the function of 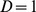 is thus to constrain the possible past states of 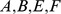 and *J*, and to constrain the possible future state of *A* (Figure 22C). Therefore, *D* = 1 specifies a concept that is exclusively self-referential to the complex to which *D* belongs (note that, in this simple version of a recurrent segment/dot system, feed-forward and feed-back connections have the same absolute strength of 1. In a more realistic neural network, in which the function of the recurrent connections is mostly modulatory, a concept's past and future purviews would be modified accordingly). Nevertheless, in this case there is a good correspondence between the intrinsic and the extrinsic perspective, since the cause repertoire of 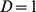 specifies as potential causes those states in which both ports-in *A* and *B* are 1, which happens when two contiguous pixels on the left are on. Importantly, the concept of 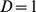 additionally takes into account the internal context 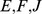 (blue shaded states in Figure 22C). However, the correspondence between intrinsic and extrinsic perspective breaks down for the ports-in 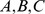: even though their state is partly determined by the external inputs, their concept specifies constraints about past and future states of elements higher up in the system, rather than about the environment (Figure 22D).

The self-referential property of the concepts specified by ports-in may have some implications with respect to the role of primary areas in consciousness. An influential hypothesis by Crick and Koch [61] suggests that primary visual cortex (V1) and perhaps other primary cortical areas may not contribute directly to consciousness, a hypothesis that is now supported by a large number of experimental results. For example, during binocular rivalry neurons in V1 may fire selectively to horizontal bars that are shown to one eye, even though the subject does not see them and is conscious of a different stimulus presented to the other eye [62]. On the other hand, the firing of units higher up in the visual system correlates tightly with the experience. While these results are compelling, other interpretations are possible if, as illustrated in the segment/dot system, V1 neurons were to constitute ports-in of the main complex. Under this assumption, V1 units would have to specify concepts about other units in the complex – either other V1 units or units in higher areas – rather than about their feed-forward inputs, which would remain outside the complex. V1 concepts could relate for example to Gestalt properties such as spatial continuity, rather than to oriented bars. In that case, what V1 contributes to consciousness during binocular rivalry – namely spatial continuity – would not change substantially between the two rivalrous percepts. Instead, concepts corresponding to oriented bars would be specified by units in higher areas, whose firing is sensitive to perceptual rivalry, *over* units in V1. In sum, V1 units would contribute to consciousness not only by generating their own concepts (such as spatial continuity), but also by providing the cause repertoire for concepts specified by units higher up (such as oriented bars). While this possibility may be far-fetched and counterintuitive, it would not be inconsistent with lesion studies that highlight the importance of V1 for most aspects of visual consciousness [63], [64].

The self-referential nature of concepts within a complex has implications with respect to how concepts obtain their *meaning*. As mentioned above, a (conscious) external observer “knows” that element *F* in Figure 22E turns on whenever there is a “segment” in the input from the environment. However, from the intrinsic perspective of the complex, that meaning cannot be specified by *F* = 1 in isolation. This is because, while the cause repertoire of *F* = 1 specifies that either *D* or *E* must have been on, by itself it cannot specify what *D* and *E* mean in turn. In fact, the full meaning of “segment” can only be synthesized through the interlocking of cause-effect repertoires of multiple concepts within a MICS (such as that of element *F* interlocked with those of elements *D*, *E*, and so on). In this view, the meaning of a concept depends on the *context* provided by the entire MICS to which it belongs, and corresponds to how it constrains the overall “shape” of the MICS. Meaning is thus both self-referential (internalistic) and *holistic*. A proper treatment of how the conceptual structure of a complex of mechanisms can give rise to meaning from the intrinsic perspective is beyond the scope of the present work and will be addressed in more detail elsewhere.

While emphasizing the self-referential nature of concepts and meaning, IIT naturally recognizes that in the end most concepts owe their origin to the presence of regularities in the environment, to which they ultimately must refer, albeit only indirectly. This is because the mechanisms specifying the concepts have themselves been honed under selective pressure from the environment during evolution, development, and learning [65]–[67]. Nevertheless, at any given time, environmental input can only act as a background condition, helping to “select” which particular concepts within the MICS will be “on” or “off”, and their meaning will be defined entirely within the quale. Every waking experience should then be seen as an “awake dream” selected by the environment. And indeed, once the architecture of the brain has been built and refined, having an experience – with its full complement of intrinsic meaning – does not require the environment at all, as demonstrated every night by the dreams that occur when we are asleep and disconnected from the world.

### Limitations and future directions

In finishing, we point out some limitations and unfinished business. IIT 3.0 starts from key properties of consciousness – the phenomenological axioms – and translates them into postulates that lay out how a system of mechanisms must be constructed to satisfy those axioms and thus generate consciousness. To be able to formulate the postulates in explicit, computable terms, we considered small systems of interconnected mechanisms that are fully characterized by their transition probability matrix (TPM). For each system, mechanisms are discrete in time and space (see also Text S2) and transition probabilities are available for every possible state. Directly applying this approach to physical systems of interest, such as brains, is unfeasible for several reasons: i) One would need either to discretize the variables of interest or to extend the theoretical treatment to continuous variables. ii) For biological systems, one is usually limited to observable system states, and the exhaustive perturbation of a system as the brain across all its possible states is unfeasible. Nevertheless, systematic perturbations of brain states using naturalistic stimuli such as movies can provide useful approximations. Also, circumscribed regions of the cerebral cortex could be perturbed systematically using optogenetic methods coupled with calcium imaging. Moreover, discrete, analytically tractable brain models based on neuroanatomical connectivity such as [68] could provide a suitable approximation of large-scale neural mechanisms yet permit the rigorous measurement of integrated information. iii) Variables recorded in most neurophysiological experiments may not correspond to the spatial and temporal grain at which integrated information reaches a maximum, which is the appropriate level of analysis [20]. iv) The present analysis is unfeasible for systems of more than a dozen elements or so. This is because, to calculate Φ^Max^ exhaustively, all possible partitions of every mechanism and of every system of mechanisms should be evaluated, which leads to a combinatorial explosion, not to mention that the analysis should be performed at every spatio-temporal grain. For these reasons, the primary aim of IIT 3.0 is simply to begin characterizing, in a self-consistent and explicit manner, the fundamental properties of consciousness and of the physical systems that can support it. Hopefully, heuristic measures and experimental approaches inspired by this theoretical framework will make it possible to test some of the predictions of the theory [14], [69]. Deriving bounded approximations to the explicit formalism of IIT 3.0 is also crucial for establishing in more complex networks how some of the properties described here scale with system size and as a function of system architecture.

The above formulation of IIT 3.0 is also incomplete: i) We did not discuss the relationship between MICS and specific aspects of phenomenology, such as the clustering into modalities and submodalities, and the characteristic “feel” of different aspects of experience (space, shape, color and so on; but see [4]–[6], [18]). ii) In the examples above, we assumed that the “micro” spatio-temporal grain size of elementary logic gates updating every time step was optimal. In general, however, for any given system the optimal grain size needs to be established by examining at which spatio-temporal level integrated information reaches a maximum [20]. In terms of integrated information, then, the macro may emerge over the micro, just like the whole may emerge above the parts. iii) While emphasizing that meaning is always internal to a complex (it is self-generated and self-referential), we did not discuss in any detail how meaning originates through the nesting of concepts within MICS (its holistic nature). iv) In IIT, the relationship between the MICS generated by a complex of mechanisms, such as a brain, and the environment to which it is adapted, is not one of “information processing”, but rather one of “matching” between internal and external causal structures [4], [6]. Matching can be quantified as the distance between the set of MICS generated when a system interacts with its typical environment and those generated when it is exposed to a structureless (“scrambled”) version of it [6], [70]. The notion of matching, and the prediction that adaptation to an environment should lead to an increase in matching and thereby to an increase in consciousness, will be investigated in future work, both by evolving simulated agents in virtual environments (“animats” [71]–[73]), and through neurophysiological experiments. v) IIT 3.0 explicitly treats integrated information and causation as one and the same thing, but the many implications of this approach need to be explored in depth in future work. For example, IIT implies that each individual consciousness is a local maximum of causal power. Hence, if having causal power is a requirement for existence, then consciousness is maximally real. Moreover, it is real in and of itself – from its own intrinsic perspective – without the need for an external observer to come into being.

## Supporting Information

Figure S1Motivation for exclusion at the level of mechanisms. Core cause: only one cause exists intrinsically – the most irreducible one. A neuron that receives two strong inputs from 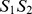 and four weak inputs 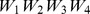. The core cause is 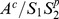 with 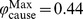 (in the case of identical 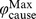 values, the largest purview is chosen because it specifies information about more system elements for the same value of irreducibility). This example illustrates that a core cause is not the most comprehensive set of possible causes of a particular state (in this case 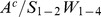), but the subset that is most affected by a partition.(PDF)

Text S1 Main differences between IIT 3.0 and earlier versions.
(PDF)

Text S2 Supplementary methods.
(PDF)

Text S3 Some differences between integrated information and Shannon information.
(PDF)
