# Supplementary material for: From the Phenomenology to the Mechanisms of Consciousness: Integrated Information Theory 3.0
Source: PLoS Comput Biol. 2014 May 8;10(5):e1003588. doi: 10.1371/journal.pcbi.1003588 (PMC4014402; doi:10.1371/journal.pcbi.1003588)
Supplement: Figure S1 — Motivation for exclusion at the level of mechanisms. Core cause: only one cause exists intrinsically – the most irreducible one. A neuron that receives two strong inputs from and four weak inputs . The core cause is with (in the case of identical values, the largest purview is chosen because it specifies information about more system elements for the same value of irreducibility). This example illustrates that a core cause is not the most comprehensive set of possible causes of a particular state (in this case ), but the subset that is most affected by a partition. (PDF) [file pcbi.1003588.s001.pdf]

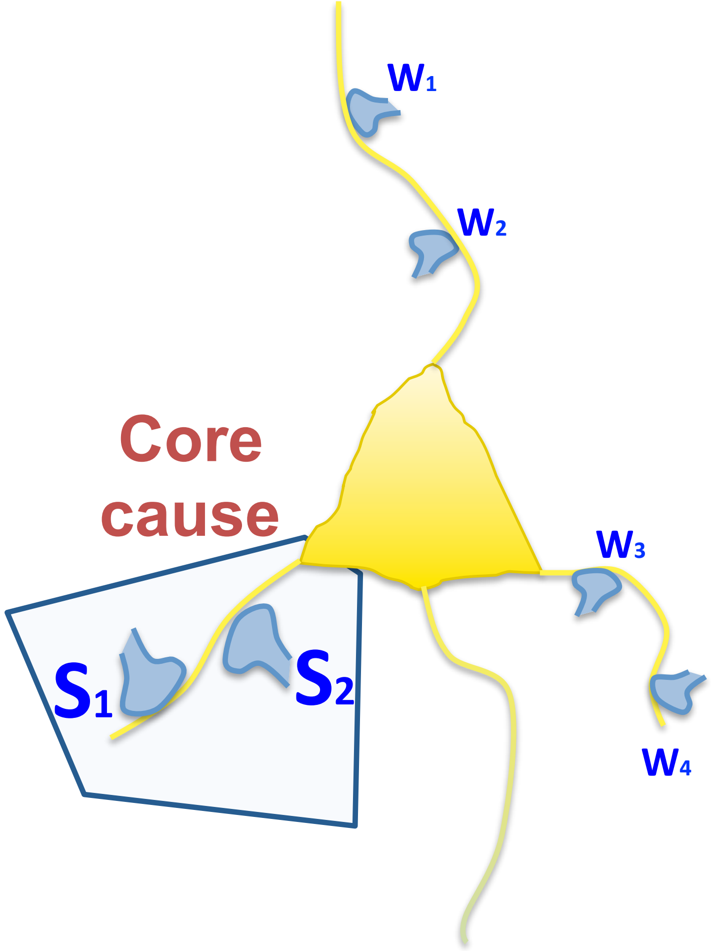

$$A^c/S_1^p \rightarrow \left(A^c/[\,]\right)\times\left([\,]/S_1^p\right) \qquad \varphi_{cause}=0.44$$

$$A^c/W_1^p \rightarrow \left(A^c/[\,]\right)\times\left([\,]/W_1^p\right) \qquad \varphi_{cause}=0.056$$

$$A^c/S_{1-2}^p \rightarrow \left(A^c/S_1^p\right)\times\left([\,]/S_2^p\right) \quad \text{Core cause} \quad \varphi_{cause}^{Max}=0.44$$

$$A^c/S_1W_1^p \rightarrow \left(A^c/S_1^p\right)\times\left([\,]/W_1^p\right) \qquad \varphi_{cause}=0.056$$

$$A^c/W_{1-2}^p \rightarrow \left(A^c/W_1^p\right)\times\left([\,]/W_2^p\right) \qquad \varphi_{cause}=0.056$$

$$A^c/S_{1-2}W_1^p \rightarrow \left(A^c/S_{1-2}^p\right)\times\left([\,]/W_1^p\right) \qquad \varphi_{cause}=0.056$$

$$A^c/S_1W_{1-2}^p \rightarrow \left(A^c/S_1W_1^p\right)\times\left([\,]/W_2^p\right) \qquad \varphi_{cause}=0.056$$

$$A^c/W_{1-3}^p \rightarrow \left(A^c/W_{1-2}^p\right)\times\left([\,]/W_3^p\right) \qquad \varphi_{cause}=0.056$$

$$\vdots \qquad \qquad \vdots$$

$$A^c/S_{1-2}W_{1-4}^p \rightarrow \left(A^c/W_1^p\right)\times\left([\,]/S_{1-2}W_{2-4}\right)^p \qquad \varphi_{cause}=0.056$$
