# Supplementary material for: From the Phenomenology to the Mechanisms of Consciousness: Integrated Information Theory 3.0
Source: PLoS Comput Biol. 2014 May 8;10(5):e1003588. doi: 10.1371/journal.pcbi.1003588 (PMC4014402; doi:10.1371/journal.pcbi.1003588)
Supplement: Text S1 — Main differences between IIT 3.0 and earlier versions. (PDF) [file pcbi.1003588.s002.pdf]

Main differences between IIT 3.0 and earlier versions

1. The axioms and postulates of the theory are presented explicitly. This clarifies many issues and highlights the link between the starting point of IIT, which is phenomenology itself (axioms that are assumed to be self-evident from the intrinsic perspective of a conscious entity) and the postulates that must be satisfied by physical systems in order to support consciousness. Moreover, the postulates are applied explicitly both at the level of individual mechanisms and at that of systems of mechanisms. As illustrated in the main text, axioms and postulates include existence, composition, information, integration, and exclusion.

2. Mechanisms specify both causes and effects. Unlike IIT 2.0 [1–3], IIT 3.0 considers how mechanisms in a state constrain both the past and the future of a system. In a way this is a return to the state-independent framework of IIT 1.0 [4], which considered both causes and effects, but only for a stationary system at equilibrium. It is also a return to IIT 1.0 in capturing the idea that information is "a difference that makes a difference", and not simply "a difference". Indeed, IIT 3.0 postulates that both cause and effect are necessary to generate information intrinsically. This emphasis on both the causes and the effects of mechanisms in a state becomes a starting point for exploring the relationship between information and causation [5], which in IIT 3.0 are one and the same thing.

3. The elements of a system are mechanisms in a state. In IIT 3.0, the basic elements that define concepts and constellations of concepts within concept space are mechanisms in a state, rather than the connections among them, as was the case in IIT 2.0. This is because mechanisms in a state (e.g. on/off) can specify "*given* this cause - *then* that effect" conditions, i.e. specify concepts.

4. Complexes are identified by assessing the effects of partitions on their entire conceptual structure. For computational expediency, in IIT 1.0 and 2.0, complexes were identified by assessing the irreducibility of a set of elements through partitions of its highest-order concept only - the concept specified by all its elements together. Only then would one establish the full conceptual structure specified by the set. In IIT 3.0, the irreducibility of a set of elements is assessed by considering how a partition affects its entire conceptual structure - all the concepts specified by its elements in all combinations (power set). In this way all the concepts that are changed or lost due to the partition contribute to Φ. For example, even a partition between a single element A and the rest of the set can destroy or modify not only the elementary concept specified by A by itself, but also the higher-order concepts A specifies together with elements on the other side of the partition, as well as all the concepts specified by other elements that include A in their purview.

5. The minimum information partition (MIP) is evaluated without normalization. In IIT 1.0 and 2.0, normalization was used to avoid certain inappropriate consequences of identifying complexes based exclusively on partitions at the level of the highest-order concept. In IIT 3.0 this is no longer necessary.

6. Mechanisms specify concepts only if they are irreducible. IIT 3.0 recognizes that concepts can only exist intrinsically if they are irreducible. This important requirement had been overlooked in IIT 2.0.

7. Concept space has a proper metric. In IIT 2.0, the effect of partitions was measured by the Kullback-Leibler divergence (KLD) between distributions, which only takes into account differences in selectivity. IIT 3.0 recognizes the need for a true metric (the earth mover's distance, EMD) that also takes into account the similarity or dissimilarity of states between whole and partitioned distributions. Moreover, an extended version of EMD is applied to measure the distance between whole and partitioned constellations of concepts in concept space. This development makes all the

more explicit the distinction between the notion of information in IIT as "differences that make a difference" from the intrinsic perspective of a system, and the classic notion of information from the perspective of an external observer (see Text S2).

8. The exclusion postulate is applied not only to systems of mechanisms but also to causes and effects specified by individual mechanisms in a system.

9. Elements outside the candidate set under consideration are treated as background conditions. Their states are fixed at their "actual" values, rather than noised (see Supplementary Methods).

10. A user-friendly program for calculating exhaustively all the quantities required by IIT in discrete systems is made available alongside the paper [6].

References

1. Tononi G (2008) Consciousness as integrated information: a provisional manifesto. Biol Bull 215: 216-242.

2. Balduzzi D, Tononi G (2008) Integrated information in discrete dynamical systems: Motivation and theoretical framework. PLoS Comput Biol 4: e1000091.

3. Balduzzi D, Tononi G (2009) Qualia: the geometry of integrated information. PLoS Comput Biol 5: e1000462.

4. Tononi G (2004) An information integration theory of consciousness. BMC Neurosci 5: 42.

5. Albantakis L, Hoel EP, Koch C, Tononi G (2013) Intrinsic Causation and Consciousness. Association for the scientific study of consciousness conference (ASSC17). Available at www.theassc.org/files/assc/docs/ASSC17-PB-070113-online-version-with-Addendum.pdf. Accessed November 2, 2013.

6. https://github.com/Albantakis/iit/tree/IIT-3.0-Program
