# Supplementary material for: From the Phenomenology to the Mechanisms of Consciousness: Integrated Information Theory 3.0
Source: PLoS Comput Biol. 2014 May 8;10(5):e1003588. doi: 10.1371/journal.pcbi.1003588 (PMC4014402; doi:10.1371/journal.pcbi.1003588)
Supplement: Text S2 — Supplementary methods. (PDF) [file pcbi.1003588.s003.pdf]

Supplementary Methods

Conditionally independent mechanisms

In this paper, it is assumed that mechanisms are conditionally independent. Consider a system consisting of three elements ABC. Mathematically, conditional independence of mechanisms A, B, and C is represented as

$$p(ABC^t|ABC^{t-1}) = p(A^t|ABC^{t-1}) \times p(B^t|ABC^{t-1}) \times p(C^t|ABC^{t-1}). \quad (1)$$

In words, given a system state at time t_{-1}, the probability of A, B, and C at time t can be determined independently. This corresponds to the assumption that there is no instantaneous interaction between mechanisms and causes must precede their effects.

Background conditions

Choosing a "candidate set" for IIT analysis means defining a precise border between elements that are inside the candidate set and elements that are outside. From the intrinsic perspective of the candidate set, the outside elements are treated as *background conditions*. This means that they are not considered as variables internal to the set over which to perform perturbations, but rather as fixed, external constraints. For the purpose of IIT analysis, this means that the connections from the outside elements are not noised and their state is kept fixed. Specifically, when evaluating a cause repertoire in the candidate set, the outside elements are fixed at their past state at t_{-1}. Similarly, when evaluating an effect repertoire, the outside elements are fixed at their present states at t_0.

As an example, in the candidate set of Fig. 1 (main text), the state of element D is taken to be 0 at t_{-1} and t_0. Given these background condition, the candidate set ABC performs as if D did not exist. On the other hand, the transition probability matrix (TPM) of ABC would be different for $D(t_{-1}) = 1$. Thus, the conceptual structure (C) of the candidate set may differ, depending on the background conditions, even though the state of the elements within the candidate set is the same (Note that, for all example systems in the Results and Discussion section, the system state at t_{-1} is the same as the current state at t_0. Also, when considering other candidate sets within ABC, it is assumed that $ABC(t_{-1}) = 110$.)

A neurological example of background conditions would be the sensory input to the ports-in of a cortical main complex. As was illustrated in the case of the segment/dot system (Fig. 22, main text), the main complex does not include the "sensory" elements providing feed-forward input to it. However, their input - some on and some off - constitutes a background condition over which cause-effect repertoires internal to the main complex are evaluated. As a more extreme example, the activity of subcortical activating systems with diffuse projections that maintain the excitability of the cortex is likely to constitute an essential background or "enabling" condition [1] for the existence of a cortical main complex. Without the activating input they provide, cortical neurons become bistable and consciousness disintegrates [2]. Nevertheless, the neural elements that provide this essential activating input to cortex are themselves likely to be excluded from the main complex [3].

Cause-effect repertoire, unconstrained repertoire p^{uc}, and partitions

To calculate the cause-effect information of a mechanism in a state over a purview, its cause-effect repertoire is compared against the unconstrained repertoire p^{uc}. The integrated information ϕ of a mechanism in a state over a purview is assessed by comparing its cause-effect repertoire against that of the partitioned purview. How these probability distributions are derived is illustrated using the example of mechanism $A = 1$ over the purview ABC from Fig. 4 (main text), as well as other mechanisms from the candidate set ABC (Fig. 1, main text).

Cause repertoire

The cause repertoire $p(ABC^p|A^c = 1)$ is obtained via Bayes rule by perturbing the set of elements into all its states with equal likelihood, i.e. assuming a uniform prior distribution $p^{\text{per}}(ABC^p)$ of past states ABC^p, where the superscript ^{per} stands for "perturbed":

$$p(ABC^p|A^c = 1) = \frac{p(A^c = 1|ABC^p)p^{\text{per}}(ABC^p)}{p(A^c = 1)}. \tag{2}$$

Here, $p^{\text{per}}(ABC^p) = 1/8$ for each past state, since the set is perturbed into each state with equal probability, $p(A^c = 1) = 3/4$, and $p(A^c = 1|ABC^p)$ is either 0 (for states 000 and 100) or 1 (for all other states).

In general, the cause repertoire can also be assessed over a subset of the candidate set, e.g. $p(C^p|A^c = 1)$. In this case, one has to *marginalize* over the elements outside of the purview, which remain unconstrained:

$$p(C^p|A^c = 1) = \frac{\left(\sum_{AB^p} p(A^c = 1|C^p, AB^p)\, p^{\text{per}}(AB^p)\right)\, p^{\text{per}}(C^p)}{p(A^c = 1)}, \tag{3}$$

where $p^{\text{per}}(AB^c)$ denotes the perturbed (uniform) distribution over the past states of the elements AB. The sum in equation 3 is effectively the average over the probabilities $p(A^c = 1|ABC^p)$ calculated for $AB^p = [00, 10, 01, 11]$.

If the cause-repertoire of a higher order mechanism such as $AB = 10$ is determined over a limited purview, e.g AB^p, marginalizing over the remaining elements (C^p) can lead to correlations in AB^c if C provides common input to both A and B. Since the aim is to assess the cause-information of AB^c about AB^p independent of C^p, C^p has to be replaced by "virtual elements" with independent output to every element, as indicated by the subscript $_V$:

$$p(AB^p|AB^c = 10) = \frac{\left(\sum_{C_V^p} p(AB^c = 10|AB^p, C_V^p)\, p^{\text{per}}(C_V^p)\right)\, p^{\text{per}}(AB^p)}{p(AB^c = 10)}. \tag{4}$$

The virtual element C_V^p means that the states 0 and 1 are imposed independently over every output connection from C (Fig. 1A). The mechanisms considered here are 1st order Markov functions and are thus conditionally independent given their respective inputs. Therefore, the cause repertoire of a higher order mechanism such as $AB = 01$ can be calculated as the product of the cause-repertoires of its elementary mechanisms, $A = 1$ and $B = 0$, obviating the need to add virtual elements in the actual calculations:

$$p(AB^p|AB^c = 10) = p(AB^p|A^c = 1) \times p(AB^p|B^c = 0). \tag{5}$$

Unconstrained cause repertoire

As described in the main text (Fig. 4), the amount of cause information that $A = 1$ specifies about the past, its cause information (ci), is measured as the distance D between the cause repertoire (eq. 2) and the unconstrained repertoire p^{uc}. For the purview ABC^p:

$$ci(ABC^p|A^c = 1) = D(p(ABC^p|A^c = 1)||p^{\text{uc}}(ABC^p)) = 0.33. \tag{6}$$

$p^{\text{uc}}(ABC^p)$ corresponds to the cause repertoire in the absence of any mechanism. The unconstrained past distributions is thus the uniform distribution, since without the constraints of a mechanism in a state $p^{\text{uc}}(ABC^p) = p^{\text{per}}(ABC^p)$.

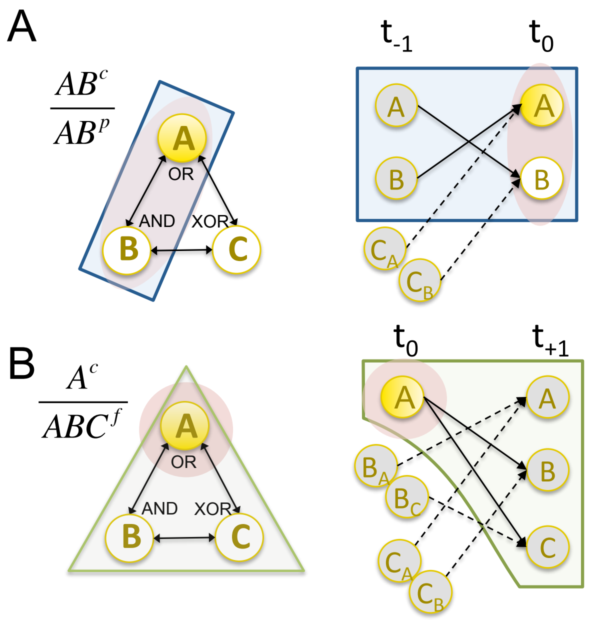

Figure 1. Virtual elements. The elements inside the candidate set but outside of the purview under consideration are replaced by virtual elements. (A) For the past purview AB^c/AB^p, element C^p is replaced by virtual elements C^p_A and C^p_B with independent outputs to A^c and B^c. This means that C^p_A and C^p_B are independently set to 0 and 1 with equal probability when the cause repertoire of AB^c/AB^p is assessed. (B) Likewise, to assess the effect repertoire of the future purview A^c/ABC^f, the two elements outside of the purview B^c and C^c are replaced by virtual elements with independent, unconstrained inputs to ABC^f.

Effect repertoire

The effect repertoire $p(ABC^f|A^c = 1)$ (Fig. 4, main text) is computed by fixing the current state of A to 1, while the remaining elements B and C are independently perturbed into all their possible states with equal likelihood:

$$p(ABC^f|A^c = 1) = \sum_{BC^c_V} p(ABC^f|A^c = 1, BC^c_V)p^{\text{per}}(BC^c_V). \quad (7)$$

Again, common inputs from B or C can lead to correlations between A^f, B^f, and C^f. To avoid counting these correlations as effects of element A, it is important to replace elements B and C by virtual elements with independent outputs to every element (Fig. 1B). Since all mechanisms under consideration are conditionally independent, in practice the effect repertoire can be calculated as:

$$p(ABC^f|A^c = 1) = p(A^f|A^c = 1) \times p(B^f|A^c = 1) \times p(C^f|A^c = 1), \quad (8)$$

where the effect repertoire of a single future element, e.g. $p(A^f|A^c = 1)$ is simply:

$$p(A^f|A^c = 1) = \sum_{BC^c} p(ABC^f|A^c = 1, BC^c)p^{\text{per}}(BC^c). \quad (9)$$

Unconstrained effect repertoire

Like the cause information (ci), the effect information (ei) of $A = 1$ is quantified as the distance D between the effect repertoire of A and the unconstrained future distribution $p^{\mathrm{uc}}(ABC^f)$:

$$ei(ABC^f|A^c = 1) = D(p(ABC^f|A^c = 1)||p^{\mathrm{uc}}(ABC^f)) = 0.25. \tag{10}$$

Without any constrains from a mechanism in a state, the unconstrained effect repertoire $p^{\mathrm{uc}}(ABC^f)$ is given by:

$$p^{\mathrm{uc}}(ABC^f) = \sum_{ABC^c_V} p(ABC^f|ABC^c_V)p^{\mathrm{per}}(ABC^c_V), \tag{11}$$

where virtual elements are again used to avoid including effects arising from correlations due to common inputs. For conditionally independent mechanisms, this is identical to:

$$\begin{aligned} p^{\mathrm{uc}}(ABC^f) = &\sum_{ABC^c} p(A^f|ABC^c)p^{\mathrm{per}}(ABC^c) \times \sum_{ABC^c} p(B^f|ABC^c)p^{\mathrm{per}}(ABC^c)\times \\ &\times \sum_{ABC^c} p(C^f|ABC^c)p^{\mathrm{per}}(ABC^c), \end{aligned} \tag{12}$$

the product of the effect probability distributions of each element given unconstrained inputs. $p^{\mathrm{uc}}(ABC^f)$ is thus not simply the uniform distribution of future states of the candidate set. In the example set ABC, the unconstrained effect repertoire for the OR-gate A is $p(A = 0) = 0.25$ and $p(A = 1) = 0.75$, for the AND-gate B: $p(B = 0) = 0.75$ and $p(B = 1) = 0.25$, and for the XOR-gate C: $p(C = 0) = 0.5$ and $p(C = 1) = 0.5$, obtained by perturbing their inputs to the states [00, 01, 10, 11] with equal probability.

Partitions

As illustrated above, when the cause/effect repertoire of a mechanism is computed over a particular purview, the elements outside of the purview, but within the candidate set, remain unconstrained. Similarly, if a purview is partitioned, elements outside of the part under consideration become unconstrained and thus effectively act as independent noise sources (they are "injected with noise"). This renders the connections across the partition causally inactive.

In the example shown in Fig. 2, the purview ABC^c/ABC^f is partitioned into $AB^c/AB^f \times C^c/C^f$. To obtain the partitioned effect repertoire, the effect repertoires of the purviews AB^c/AB^f and C^c/C^f are calculated independently and then multiplied. Considering the purview of AB^c/AB^f, the element C is outside of the purview and thus unconstrained:

$$p(AB^f|AB^c = 10) = \sum_{C^c_V} p(AB^f|AB^c = 10, C^c_V)p^{\mathrm{per}}(C^c_V). \tag{13}$$

To causally disconnect C from AB, it is again important to introduce virtual elements, which ensure independent noise in the connections across the partition. Even if A and B receive a common input from C, such a common input is ignored in the purview of AB^c/AB^f. Since A, B, and C are conditionally independent $p(AB^f|AB^c = 10)$ can be calculated without explicitly introducing virtual units:

$$p(AB^f|AB^c = 10) = \sum_{C^c} p(A^f|AB^c = 10, C^c)p^{\mathrm{per}}(C^c) \times \sum_{C^c} p(B^f|AB^c = 10, C^c)p^{\mathrm{per}}(C^c). \tag{14}$$

Similarly, the purview of C^c/C^f is the computed as:

$$p(C^f|C^c = 0) = \sum_{AB^c} p(C^f|C^c = 0, AB^c)p^{\mathrm{per}}(AB^c). \tag{15}$$

The partitioned effect repertoire, $AB^c/AB^f \times C^c/C^f$, is the product of $p(AB^f|AB^c)$ and $p(C^f|C^c)$. Partitioned cause repertoires are calculated in the same way.

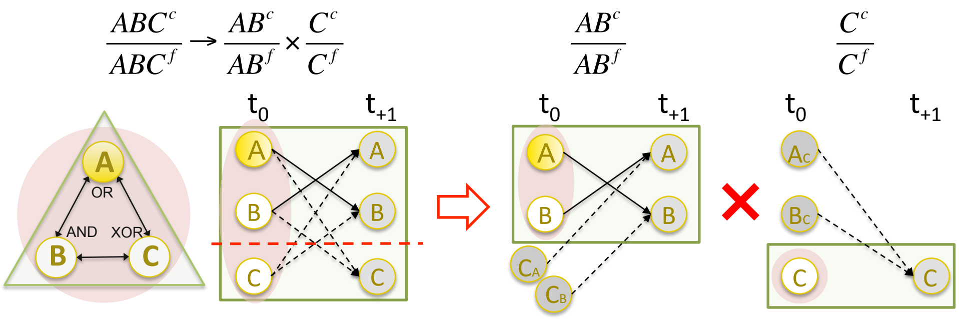

Figure 2. Partitioning a purview. The purview ABC^c/ABC^f is partitioned into $AB^c/AB^f \times C^c/C^f$. Connections outside of each partition are "injected with noise". This means that the elements outside of the respective part AB^c/AB^f and C^c/C^f are replaced by virtual elements with independent, unconstrained outputs. The partitioned effect repertoire is the product of the effect repertoires of the parts.

Earth mover's distance

Distance for probability distributions

Integrated information φ measures the difference between two probability distributions, a partitioned distribution and an unpartitioned distribution. In previous work, the Kullback-Leibler divergence (KLD) was used to compare distributions. KLD has several useful properties, but it is not a true metric (it is not symmetric) and it is unbounded. Moreover, KLD only measures how "sharp" a distribution is compared to the other, without taking into account whether some states of the system are closer than others (e.g. that [0 0] is closer to [1 0] than to [1 1]).

A more appropriate measure that corresponds better to the IIT notion of information as "differences that make a difference" is the earth mover's distance (EMD). As indicated by its name, an intuitive interpretation of the EMD is that it measures the minimum cost of transportation that arises when one probability distribution has to be transformed into another [5, 6]. In this view, a probability value is associated with a certain amount of "earth" that is moved across a certain distance, the distance from one state to another. The cost of transportation is then the amount of "earth" moved times the distance by which it is moved. The distance between binary states is measured by the Hamming distance, which counts the number of places by which two strings differ. For instance, the Hamming distance between the states $ABC = 000$ and $ABC = 111$ is 3; the distance between $ABC = 010$ and $ABC = 100$ is 2. The EMD is in principle extendable to account for non-binary states, as long as a distance between the individual states is given, which is an intrinsic property of the mechanisms under consideration.

EMD is symmetric, is bounded (by the number of elements N for binary mechanisms) and takes into account the distance between states. Fig. 3 shows a cause repertoire over two elements with two of its possible partitioned repertoires. In the intact cause repertoire, the only possible cause is 00. Partitions 1 and 2 both have state 00 as a possible cause, but add state 10 (Partition 1) and state 11 (Partition 2), with equal probability. Since state 00 is closer to 10 than to 11, the intact distribution is closer to Partition 1 than to the Partition 2, as captured with EMD. From the intrinsic perspective of the system, then, Partition 2 makes more of a difference, since the state of *two* system mechanisms becomes undetermined instead of just one. By contrast, KLD assigns the same distance to both partitions, since it only measures the "reduction of uncertainty."

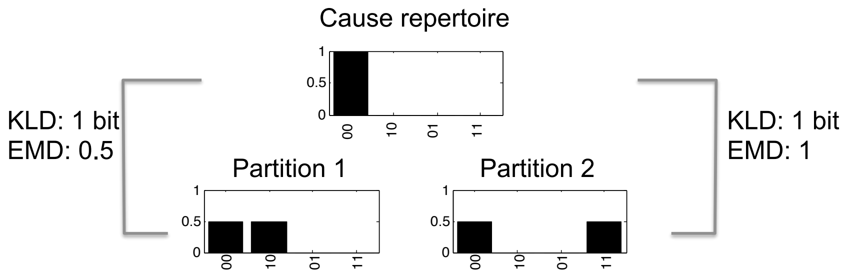

Figure 3. Earth Mover's Distance EMD, but not Kullback-Leibler Distance KLD, takes into account distances between states. A cause repertoire and two of its possible partitions are shown. KLD assigns the same value to both partitions, since it only measures the reduction of uncertainty of the cause repertoire with respect to the partitions. By contrast, EMD is sensitive to the distance between states. Since 00 is less distant from 10 than from 11, in terms of EMD Partition 2 makes more of a difference than Partition 1.

Distance for constellations of concepts

Integrated conceptual information Φ^{Max} measures the difference between the intact constellation of a set of elements C and that of its minimum information partition $C_{\rightarrow}^{\text{MIP}}$. Like the difference between probability distributions φ, the difference between constellations is assessed by an extended version of the earth mover's distance (EMD). The extended EMD measures the minimal cost of transforming one constellation into another. Instead of probabilities, in the extended EMD it is the φ value of the concepts that corresponds to the "earth" that is redistributed from constellation C to $C_{\rightarrow}^{\text{MIP}}$. Instead of the Hamming distance, the distance between the concepts of C and $C_{\rightarrow}^{\text{MIP}}$ is given by their distance in concept space. Since $\sum(\varphi^{\text{Max}})$ of all concepts of C is usually higher than that of $C_{\rightarrow}^{\text{MIP}}$, any residual φ^{Max} is assigned to the "null" concept (the unconstrained distribution p^{uc}), which is included as an additional location in $C_{\rightarrow}^{\text{MIP}}$.

Fig. 4 shows how the Φ^{Max} value of candidate set ABC (Fig. 12, main text) is calculated. To illustrate the analogy between the standard EMD for probability distributions and the extended EMD for constellations, the φ^{Max} values of the concepts of C and $C_{\rightarrow}^{\text{MIP}}$ are displayed as two distributions. In this example, the concept of A and B are unaffected by the partition, while the other 4 concepts are destroyed. The optimal way to transform C into $C_{\rightarrow}^{\text{MIP}}$ is thus to move the φ^{Max} values of the concepts C, AB, BC, and ABC to the "null" concept p^{uc}. The distance between two concepts in concept space is measured by the EMD distance of their cause-effect repertoires. The distance from concept C to the "null" concept, for example (Fig. 4B), is the sum of the standard EMD distance of the two cause repertoires and the two effect repertoires of C and the p^{uc}. To obtain Φ^{Max} for this example, the distances of concepts C, AB, BC, and ABC to the "null" concept are multiplied by the φ^{Max} value of each concept and then summed up. In the general case, the optimal way to redistribute φ^{Max} form C to $C_{\rightarrow}^{\text{MIP}}$ must be found using an optimization algorithm [5, 6].

Custom-made MATLAB software was used for all calculations. The program to calculate the complex of a small system of logic gates and its constellation of concepts is available under: [7]. EMD calculations were performed using the open source fast MATLAB code of Pele and Werman [5, 6].

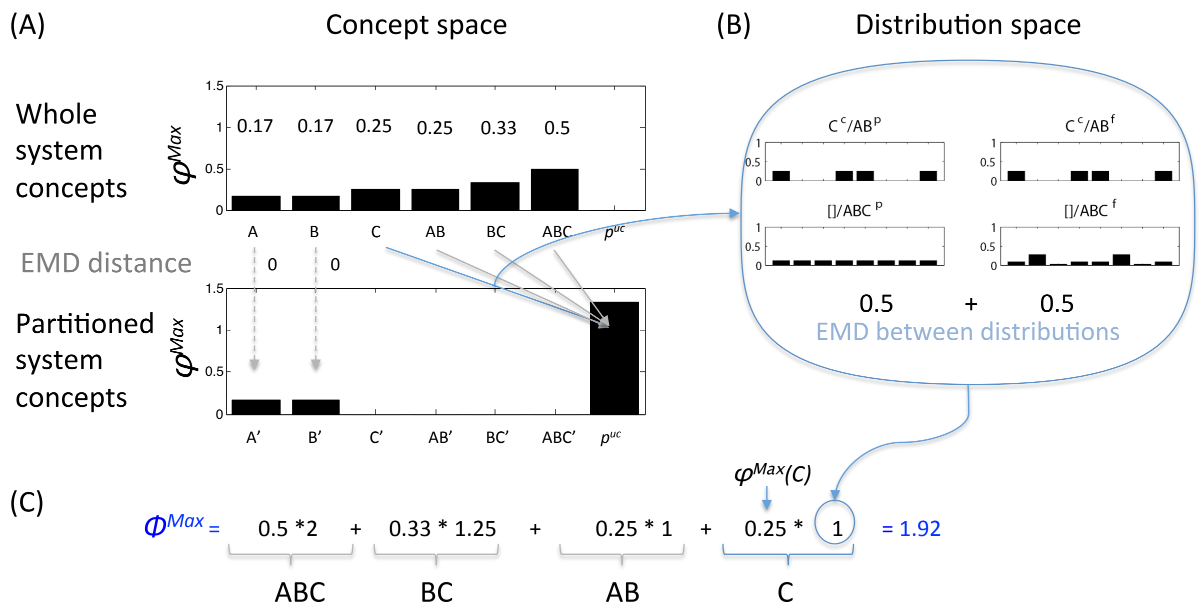

Figure 4. Extended earth mover's distance EMD in concept space. The extended EMD measures the distance between the concept constellation C of the whole set ABC and the constellation $C_{\rightarrow}^{\mathrm{MIP}}$ of the unidirectionally partitioned set (see Fig. 12, main text). (A) The concepts of C and $C_{\rightarrow}^{\mathrm{MIP}}$ from the candidate set ABC are plotted as distributions. The height of the bars corresponds to the amount of φ^{Max} of each concept. As for probability distributions, the EMD quantifies the minimal cost of transforming one φ^{Max}-distribution into the other. Since $\sum(\varphi^{\mathrm{Max}})$ of C is typically larger than that of $C_{\rightarrow}^{\mathrm{MIP}}$, any residual φ^{Max} is assigned to the "null" concept, the unconstrained cause-effect repertoire p^{uc}, in $C_{\rightarrow}^{\mathrm{MIP}}$. The distance between concepts in concept space is the EMD distance of their cause-effect repertoires. In the set ABC, the concepts of mechanisms A and B are not affected by the partition ($A = A'$ and $B = B'$). Therefore, the distance between A and A' and B and B' is 0. All other concepts are destroyed by the partition. Their φ^{Max} values are thus transported to p^{uc}. (B) The distance from concept C, for example, to p^{uc} is the sum of the EMD values obtained by comparing the cause-repertoire of C with $p^{\mathrm{uc,p}}$ and its effect repertoire with $p^{\mathrm{uc,f}}$. (C) The distance between concept C and p^{uc} is then multiplied by the difference in φ^{Max}, where $\varphi(p^{\mathrm{uc}}) = 0$ in C. Adding these values for all concepts, one obtains the Φ^{Max} value of set ABC. In the general case, calculating EMD involves optimizing the overall distance between all concepts.

References

1. Mormann F, Koch C (2007) Neural Correlates of Consciousness. Scholarpedia 2:1740.

2. Casali AG, Gosseries O, Rosanova M, Boly M, Sarasso S, et al. (2013) A theoretically based index of consciousness independent of sensory processing and behavior. Science translational medicine 5(198): 198ra105-198ra105.

3. Balduzzi D, Tononi G (2008) Integrated information in discrete dynamical systems: Motivation and theoretical framework. PLoS Comput Biol 4: e1000091.

4. Tononi G (2008) Consciousness as integrated information: a provisional manifesto. Biol Bull 215: 216-242.

5. Pele O, Werman M (2008) A linear time histogram metric for improved sift matching. Computer Vision-ECCV 2008, 495-508. Springer Berlin Heidelberg.

6. Pele O, Werman M (2009) Fast and robust earth mover's distances. Computer vision, 2009 IEEE 12th international conference on, 460-467. IEEE.

7. https://github.com/Albantakis/iit/tree/IIT-3.0-Program
