# Supplementary material for: From the Phenomenology to the Mechanisms of Consciousness: Integrated Information Theory 3.0
Source: PLoS Comput Biol. 2014 May 8;10(5):e1003588. doi: 10.1371/journal.pcbi.1003588 (PMC4014402; doi:10.1371/journal.pcbi.1003588)
Supplement: Text S3 — Some differences between integrated information and Shannon information. (PDF) [file pcbi.1003588.s004.pdf]

## Some differences between integrated information and Shannon information

While the notion of information plays a central role in IIT, it is distinctively different from that developed in Shannon's communication theory [1, 2].

Information as defined in information theory quantifies how accurately input signals can be decoded by knowing output signals transmitted across a (noisy) channel. (Mutual) information is the average statistical dependence between two sets of variables, such as the inputs and the outputs of a channel. In general, this depends on the entropy of the inputs and on how well the inputs predict the outputs. The (self)-information contained in a single system state is simply the negative logarithm of its probability of occurrence. As clearly recognized by Shannon, the information of information theory is divorced from meaning: the sender and the receiver are assumed to know what the messages mean, and information theory is only concerned with how reliably and efficiently they can be transmitted across a channel. While Shannon information can be evaluated between past states and current states of the same system, it is always assessed from the extrinsic perspective of an observer who assesses the statistical dependence between inputs and outputs. Therefore, Shannon information could be called *extrinsic* information.

In IIT, by contrast, information is *intrinsic*: it is assessed from the intrinsic perspective of a system in terms of the differences that make a difference to it. Intrinsic information is *causal*, and it must be evaluated by perturbing a set of elements in all possible ways, not just by observing them. It is also *compositional*, in that different combinations of elements within a system can simultaneously specify different constraints. Crucially, in IIT information must also be *integrated*: if partitioning a system makes no difference to it, there is no system to begin with. Moreover, IIT only considers information that is maximally integrated or *exclusive* because, from the intrinsic perspective, there can only be one set of causes. None of these requirements matter from the extrinsic perspective of an observer who measures the overall statistical dependence between inputs and outputs. Note also that within the framework of Shannon information, the difference between two probability distributions is assessed based on the Kullback-Leibler divergence (KLD), which measures the loss of Shannon information from the perspective of an external observer if one probability distribution is approximated by another. In the case that a distribution is evaluated against maximum entropy (all states equally likely), KLD measures reduction of uncertainty. Since information in IIT aims to capture differences that make a difference from the intrinsic perspective of a system, it uses a true metric (EMD) that is sensitive to the relative distance among system states (Supplementary Methods).

Most importantly, classical information theory does not deal with meaning, but only with how well messages are communicated and stored. In IIT, instead, information *is* meaning: more precisely, intrinsic integrated information is a maximally irreducible information structure (MICS). This integrated information structure captures all the constrains over the past and future of a complex as determined by the state of its mechanisms. It is thus a shape in concept space, not a message transmitted across a channel. Accordingly, the meaning is the MICS itself, and the meaning of each individual concept within the MICS is self-generated, self-referential, and holistic: it is constructed by the elements of the complex, over the elements of the complex, and in the context provided by other concepts within the same MICS.

As an example, consider the firing of a face neuron deep inside the brain that is part of the main complex. From the extrinsic perspective of an observer, such a face neuron can certainly convey extrinsic, Shannon information about some events in the environment, given that its firing is tightly correlated with the presence of faces. From the intrinsic perspective, however, the firing of that neuron is meaningful because of the way it modifies the shape of the MICS generated by the main complex (which it would modify in a different way if it were silent). As a corollary, dreams have as much meaning as awake experiences: the firing of a face neuron in a dream specifies the same intrinsic information even though it conveys no extrinsic information about the environment.

Ultimately, of course, the circuits that generate meaning originate, develop, and refine through a long process of evolution, neural development, and learning, under the selective pressure of a complex environment. According to IIT, the goal of these circuits is not so much to process extrinsic information from

the environment. Instead, it is to generate integrated information structures that "match" regularities in the environment in such a way that the "right dream" occurs at the right time [3, 4]. This matching, in turn, can act as a driving force for the evolution of consciousness [3].

# References


1. Shannon CE, Weaver W (1949) The mathematical theory of communication (University of Illinois press, Urbana, IL).

2. Cover TM, Thomas JA (2006) Elements of information theory (Wiley-interscience).

3. Tononi G (2012) Integrated information theory of consciousness: an updated account. Arch Ital Biol 150: 56-90.

4. Hashmi A, Nere A, Tononi G (2013) Sleep-Dependent Synaptic Down-Selection (II): Single-Neuron Level Benefits for Matching, Selectivity, and Specificity. Front Neurol 4:148.
